# Applicability of transferable multipole pseudo-atoms for restoring inner-crystal electronic force density fields. Chemical bonding and binding features in the crystal and dimer of 1,3-bis­(2-hy­droxy­ethyl)-6-methyl­uracil

**DOI:** 10.1107/S2052252523007108

**Published:** 2023-09-01

**Authors:** Alina F. Saifina, Sergey V. Kartashov, Liliya F. Saifina, Robert R. Fayzullin

**Affiliations:** a Arbuzov Institute of Organic and Physical Chemistry, FRC Kazan Scientific Center, Russian Academy of Sciences, 8 Arbuzov Street, Kazan 420088, Russian Federation; Formby, Liverpool, United Kingdom

**Keywords:** bond theory, charge transfer, force-field pseudoatom, quantum chemical topology, quantum crystallography

## Abstract

We considered it timely to test the applicability of transferable multipole pseudo-atoms for restoring inner-crystal electronic force density fields. The procedure was carried out on the crystal of 1,3-bis­(2-hy­droxy­ethyl)-6-methyl­uracil, and some derived properties of the scalar potential and vector force fields were compared with those obtained from the experimental multipole model and from the aspherical pseudo-atom model with parameters fitted to the calculated structure factors. The procedure was shown to accurately replicate the general vector-field behavior, the peculiarities of the quantum potentials and the characteristics of the force-field pseudoatoms, such as charge, shape and volume, as well as to reproduce the relative arrangement of atomic and pseudoatomic zero-flux surfaces along internuclear regions. It was found that, in addition to the quantum-topological atoms, the force-field pseudoatoms are spatially reproduced within a single structural fragment and similar environment. In addition, the classical and nonclassical hydrogen bonds in the uracil derivative crystal, as well as the H⋯O, N⋯O and N⋯C interactions in the free π-stacked dimer of the uracil derivative molecules, were studied using the potential and force fields within the concepts of interatomic charge transfer and electron lone pair donation–acceptance. Remarkably, the nitro­gen atoms in the N⋯O and N⋯C interactions behave rather like a Lewis base and an electron contributor. At the same time, the hydrogen atom in the H⋯O interaction, being a Lewis acid, also participates in the interatomic electron transfer by acting as a contributor. Thus, it has been argued that, when describing polar interatomic interactions within orbital-free considerations, it makes more physical sense to identify electronegative (electron occupier) and electropositive (electron contributor) atoms or subatomic fragments rather than nucleophilic and electrophilic sites.

## Abstract 2

A binding approach based on electronic force density fields was used to describe the structures of the molecule, dimer and crystal of a uracil derivative. Transferable multipole pseudo-atoms were successfully used to reconstruct the inner-crystal electronic force density fields.

## 1. Introduction

Atomic theory underlies chemistry, crystallography and materials science. Not surprisingly, attempts have been made to define an atom in a molecule or a crystal, as well as to determine a molecule in a noncovalently bonded associate or a crystal. Thus, the following questions arise: (i) How can the boundary of an atom or a molecule be described in a consistent manner (if possible)? (ii) What information is contained in an atom and its shape and internal structure? (iii) What is the mechanism of bonding (energy-based approach) and binding (force-based approach) of atoms to each other? And so on. It is worth mentioning that chemists tend to describe electron–nuclear systems, their transformations and the chemical bonding within them in the comprehensible three-dimensional physical space. There are therefore various concepts for partitioning the three-dimensional space into atomic- or molecule-like fragments, including the atomic Voronoi–Dirichlet polyhedron (Niggli, 1927 ▸), the Wigner–Seitz cell (Wigner & Seitz, 1933 ▸), the van der Waals surface (Corey & Pauling, 1953 ▸), the Hirshfeld surface (Spackman & Byrom, 1997 ▸) and so on.

Remarkably, one of the earliest attempts to identify atoms in crystals was based on the electron density (ED) distribution (Bragg *et al.*, 1922 ▸), which emphasizes the historical and conceptual proximity of quantum mechanics and crystallography (Macchi, 2020 ▸). Thereupon, the function of the ED ρ(**r**) occupies a special place. For a given arrangement of nuclei, the ED uniquely determines the Hellmann–Feynman forces of electrostatic nature, acting on the nuclei (Hellmann, 2015 ▸; Feynman, 1939 ▸). According to the Hohenberg–Kohn proof of existence (Hohenberg & Kohn, 1964 ▸), the sum of the kinetic energy of electrons and the nonclassical energy of the electron–electron interaction is the universal ED functional, and the exact ED of the ground state provides the minimum of this functional. Thus, the ED is informative enough to completely describe the ground state of an electron–nuclear system. Importantly, the inner-crystal ED *image* can be reconstructed by implementing the multipole (aspherical pseudo-atom) structural model from experimental diffraction data corrected for absorption, thermal diffuse scattering, multiple scattering and extinction (Konobeevskii, 1951 ▸; DeMarco & Weiss, 1965 ▸; Dawson, 1967 ▸; Kurki-Suonio, 1968 ▸; Hirshfeld, 1971 ▸; Stewart, 1976 ▸; Coppens & Hansen, 1977 ▸; Price & Maslen, 1978 ▸; Hansen & Coppens, 1978 ▸; Parini *et al.*, 1985 ▸; Tsirelson & Ozerov, 1996 ▸; Coppens, 1997 ▸). It is shown that the quasi-static ED image modeled by the multipole parameters is close to the quantum mechanical ED derived by the variational principle, despite the limited resolution and incomplete thermal deconvolution.

The ED defines the corresponding scalar field in three-dimensional space. The field, in turn, can be described by a set of elements, such as critical points (CPs) where the ED gradient vanishes, ∇ρ(**r**) = 0 [*e.g.* maximum CPs (3, −3) coinciding with the position of the nuclei and saddle CPs (3, −1) or bond CPs (BCPs) between the pairs of presumably chemically bonded atoms], gradient lines or, in other words, trajectories (*e.g.* a bond path consisting of two trajectories, each starting from the BCP and ending at the nucleus of one of the two bonded atoms) and zero-flux surfaces (ZFSs) *S*(Ω), which divide regions Ω with the regular pattern of trajectories:

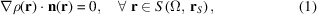

where **n**(**r**) is the unit vector orthogonal to the boundary surface *S*(Ω). Such a region or atomic ρ-basin contains a single extremum CP (3, −3), which performs as an attractor for the enclosed gradient lines. Its combination with the nucleus is defined by Bader and coworkers as the chemically bonded ∇ρ-atom or atom in a molecule (Bader & Nguyen-Dang, 1981 ▸; Bader *et al.*, 1981 ▸; Bader, 1990 ▸, 1991 ▸, 2001 ▸). Note that each bonded ∇ρ-atom is situated in the field of other ∇ρ-atoms. The ZFS *S*(Ω) is a necessary boundary condition for applying Schwinger’s principle of stationary action to define the physics of a bonded ∇ρ-atom as an open quantum system. Moreover, since equation (1) is true, it has been shown that the virial theorem (Bader, 1980 ▸) and the Hohenberg–Kohn theorem (Bader & Becker, 1988 ▸) hold for bonded ∇ρ-atoms. A characteristic feature of atoms in molecules is the transferability of various physical properties (Bader & Beddall, 1971 ▸; Bader *et al.*, 1987 ▸). The difference between the electron populations of a free atom of a chemical element and a corresponding ∇ρ-atom confined by a ZFS *S*(Ω) is the atomic charge *q*_∇ρ_(Ω) of this ∇ρ-atom. The mechanism of charge transfer occurring as a result of the formation of some simple hydrogen-bonded complexes in the context of the quantum theory of atoms in molecules (QTAIM) has been discussed in the literature (Cheeseman *et al.*, 1988 ▸; Carroll & Bader, 1988 ▸; Koch & Popelier, 1995 ▸).

The quantum-topological approach to the partitioning of many-electron multinuclear systems (Martín Pendás *et al.*, 2013 ▸; Tsirelson & Stash, 2020 ▸; Shteingolts, Stash *et al.*, 2021 ▸; Saifina *et al.*, 2023 ▸) is not limited to considering only the ED field. There are also physically valid and operative scalar one-electron potential fields and derived one-electron force density fields in crystals (Tsirelson & Stash, 2020 ▸; Shteingolts, Stash *et al.*, 2021 ▸) and free molecules and noncovalently bonded associates (Kartashov *et al.*, 2023 ▸; Saifina *et al.*, 2023 ▸; Takebayashi *et al.*, 2023 ▸). These fields reflect the natural laws of electron behavior within electron–nuclear systems (Tsirelson & Stash, 2020 ▸), allow the actual structure of the latter to be described (Shteingolts *et al.*, 2022 ▸; Saifina *et al.*, 2023 ▸), and can thus play the role of mediator between quantum mechanics and chemical structure. Examples include the electrostatic potential φ_es_(**r**), the total static potential or potential acting on an electron in a molecule (PAEM) φ_em_(**r**) (Yang & Davidson, 1997 ▸; Zhao & Yang, 2014 ▸), and the kinetic potential φ_*k*_(**r**) (Tsirelson & Stash, 2020 ▸), as well as the corresponding one-electron electrostatic force 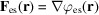 , the total static force or force acting on an electron in a molecule (FAEM) 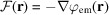 , and the total kinetic force 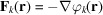 . Importantly, the kinetic and static quantum effects compensate each other in equilibrium, *i.e.* 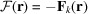 .

By analogy with the QTAIM (Bader, 1990 ▸), both in periodic crystals (Tsirelson *et al.*, 2000 ▸; Tsirelson & Stash, 2020 ▸; Shteingolts, Stash *et al.*, 2021 ▸; Stash *et al.*, 2021 ▸; Shteingolts, Voronina *et al.*, 2021 ▸; Shteingolts *et al.*, 2022 ▸; Kartashov *et al.*, 2023 ▸; Saifina *et al.*, 2023 ▸; Takebayashi *et al.*, 2023 ▸) and in nonperiodic molecules, noncovalently bonded associates, ion pairs, coordination complexes, or even models of initial and transition states (Kartashov *et al.*, 2023 ▸; Saifina *et al.*, 2023 ▸; Takebayashi *et al.*, 2023 ▸), the ZFSs *U*(Ω) and *P*(Ω) framing the φ_es_- and φ_*k*_-basins can be obtained as solutions to the following equations:

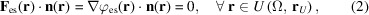




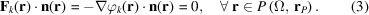

Just as the idea of zero flux in the ED gradient vector field ∇ρ(**r**) through some interatomic boundary *S*(Ω) leads to the concept of a bonded ∇ρ-atom (as well as an atomic ρ-basin), so the idea of zero flux in the electrostatic or kinetic force field, **F**_es_(**r**) or **F**_*k*_(**r**), through the other interpseudoatomic boundary *U*(Ω) or *P*(Ω) allows us to define the electrostatic or kinetic force field pseudoatom (as well as the respective pseudoatomic φ_es_- or φ_*k*_-basin) as part of a many-electron multinuclear system (Shteingolts *et al.*, 2022 ▸; Kartashov *et al.*, 2023 ▸; Saifina *et al.*, 2023 ▸). Thus, the partitioning of a many-electron multinuclear system in the position space into force-field pseudoatoms of the same type is an opportune and physically sound procedure with clear and straightforward underlying principles. Note that each pseudoatom in a multinuclear system is situated in the field of other pseudoatoms of the same type. Note that the **F**_*k*_-pseudoatoms are expected to behave similarly to the pseudoatomic regions derived from the Ehrenfest force field (Martín Pendás & Hernández-Trujillo, 2012 ▸; Shteingolts *et al.*, 2022 ▸) but accessible from the ED by applying available orbital-free density functional theory approximations (Stash & Tsirelson, 2022 ▸).

**Figure 1:**
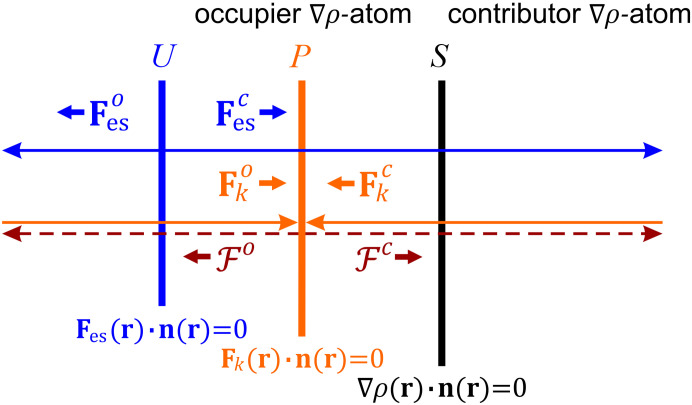
Schematic representation of the relative arrangement of the pseudo­atomic and atomic boundaries *U*(Ω), *P*(Ω) and *S*(Ω) in an internuclear space and the direction of the electronic electrostatic, kinetic and total static forces, **F**_es_(**r**), **F**_*k*_(**r**) and 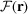 , in the regions between the boundaries. The atomic boundary *S*(Ω) is shown as a black vertical line, while the pseudoatomic boundaries *U*(Ω) and *P*(Ω) are shown as blue and orange vertical lines, respectively. The superscripts *o* and *c* indicate the force origin: electron occupier (left) and electron contributor (right), respectively, which are demarcated by *S*(Ω).

An atomic nucleus coincides with the position of the point attractors of the atomic ρ-basin and the associated pseudo­atomic φ_es_- or φ_*k*_-basins, *i.e.* CPs (3, −3) in ρ(**r**), φ_es_(**r**) and φ_*k*_(**r**), which makes it possible to determine the common filiation of the basins of a different nature (Shteingolts *et al.*, 2022 ▸). There could also be a saddle CP (3, −1) in φ_es_(**r**) or φ_*k*_(**r**) between some pairs of **F**_es_- or **F**_*k*_-pseudoatoms and a binding φ_em_- or φ_*k*_-path (*i.e.* an analog of a bond ρ-path) connecting a CP (3, −1) with two nearby CPs (3, −3). However, because of the difference in the physical sense, on the one hand, and in the tangible embodiment in three-dimensional space, on the other hand, the volume, shape and charge of the **F**_es_- and **F**_*k*_-pseudoatoms and the ∇ρ-atom differ in most cases. Therefore, their boundaries *U*(Ω), *P*(Ω) and *S*(Ω) often do not coincide, which, in turn, leads to the presence of volumetric overlapping gaps in the vicinity of the interatomic region between φ- and ρ-basins related to the neighboring nuclei. Fig. 1 ▸ shows the main features in the arrangement of the ZFSs and the direction of the forces within an internuclear region. A detailed description of the above is given by Shteingolts *et al.* (2022 ▸).

So, the gap between the φ_es_- and ρ-basins implies a picture of electrostatic interaction between covalently and noncovalently bonded atoms, and even those that are not bonded at all (Tsirelson *et al.*, 2009 ▸; Shishkina *et al.*, 2013 ▸; Bartashevich, Matveychuk *et al.*, 2014 ▸; Bertolotti *et al.*, 2014 ▸; Bartashevich, Yushina *et al.*, 2014 ▸; Shishkina *et al.*, 2010 ▸; Shteingolts, Stash *et al.*, 2021 ▸; Bartashevich *et al.*, 2021 ▸; Shteingolts, Voronina *et al.*, 2021 ▸; Shteingolts *et al.*, 2022 ▸; Kartashov *et al.*, 2023 ▸; Saifina *et al.*, 2023 ▸; Takebayashi *et al.*, 2023 ▸). Namely, within the gap, regardless of the nature of a contact, the ED belonging to the ∇ρ-atom falls into the **F**_es_-pseudoatom related to the neighboring nucleus and is consequently drawn toward or attracted to this nucleus by the force **F**_es_(**r**) emanating from this pseudoatom (Fig. 1 ▸), thus reflecting the net electrostatic interatomic attraction for polar contacts (Tsirelson *et al.*, 2009 ▸; Mata *et al.*, 2012 ▸; Shteingolts *et al.*, 2022 ▸; Saifina *et al.*, 2023 ▸). In other words, the ED of an atom appearing in the ρ-to-φ_es_-basin penetration gap is electrostatically attracted to the nucleus of the neighboring atom. Importantly, this ED forms the considered ∇ρ-atom and constitutes its charge (Shteingolts, Stash *et al.*, 2021 ▸; Saifina *et al.*, 2023 ▸).

There are some other substantial works in the literature that deal with the understanding of the relative position of the ZFS *U*(Ω) and the gap between the φ_es_- and ρ-basins during the formation of bonded systems. For instance, the gaps in question were interpreted as the so-called electrostatic attraction regions, which were used in an attempt to explain the formation of some anionic hydrogen-bonded dimers in the gas phase (Mata *et al.*, 2012 ▸, 2015 ▸) or polyiodide chains (Lamberts *et al.*, 2016 ▸). Considering the theoretical topology of ρ(**r**) and φ_es_(**r**) along the N⋯H hydrogen bonds, Mata, Molins, Alkorta & Espinosa (2007 ▸) pointed out that the region between the boundaries *U*(Ω) and *S*(Ω) is associated with the electron lone pair (LP) of the nitrogen atom and presents the respective negative charge distribution. The authors describe the case of hydrogen bonding as follows: the hydrogen-bond acceptor (oxygen or nitro­gen) atom appears partially in the so-called electrophilic influence zone (*vide infra*) of the hydrogen nucleus. However, it remains unclear to us whether the electrophilicity of the influence zone here presupposes that the hydrogen atom accepts (occupies) the electrons within the gap. If not, what is the expression of the electrophilicity? A possible answer to our question is that the atom carrying the electrophilic zone is expected to be a Lewis acid. Apparently, such an interpretation cannot be universal; however, it could be applied *ad hoc*. Therefore, we have recently proposed to use the physically meaningful terms *electron occupier* for an electronegative atom that accepts electrons in the process of interatomic charge transfer (see below) and *electron contributor* for an electropositive atom that donates electrons (Shteingolts, Stash *et al.*, 2021 ▸). It turns out that the former often acts as a Lewis base and the latter often acts as a Lewis acid (Saifina *et al.*, 2023 ▸).

Developing the ideas of Mata *et al.* (2007 ▸) that CPs (3, −1) or (3, +1) in φ_es_(**r**) (if present) help indicate the electrophilic or nucleophilic influence zones for free molecules, respectively, Bartashevich *et al.* (2019 ▸) proposed a convenient criterion for categorizing noncovalent interactions, according to which the ZFS *U*(Ω) is situated closer to the nucleus of the atom that donates electrons and delivers its nucleophilic site for bonding, whereas the ZFS *S*(Ω) lies closer to the nucleus of the atom that acts as an acceptor of electrons and provides the electrophilic site. In fact, this criterion often makes it possible to distinguish between the Lewis base (*i.e.* an LP donor), inside whose atom the boundary *U*(Ω) passes, and the Lewis acid (*i.e.* an LP acceptor) for noncovalent interactions (Kartashov *et al.*, 2023 ▸; Saifina *et al.*, 2023 ▸). However, there is a formal exception to this rule (Shteingolts, Stash *et al.*, 2021 ▸), namely the [C=]O⋯N(*sp* ^2^) interaction in the 1,6-di­methyl-3-(prop-2-yn-1-yl)pyrimidine-2,4(1*H*,3*H*)-dione crystal; new exceptions are listed in this article. Furthermore, it remains controversial what exactly the authors of the criterion mean by donating and accepting electrons. It can be argued that the information about the charge transfer path from one bonded atom to another as a result of a chemical transformation is, strictly speaking, hidden in view of the relative arrangement of the pseudoatomic and atomic boundaries, since this arrangement does not depend on the way a system is obtained (Saifina *et al.*, 2023 ▸).

Let us now turn to the physical nature of the phenomenon. Since each **F**_es_-pseudoatom behaves as an electrostatically neutral bounded pseudoatomic region mimicking a neutral atom distorted by the inner-crystal electric field (Tsirelson *et al.*, 2000 ▸; Shteingolts *et al.*, 2022 ▸; Kartashov *et al.*, 2023 ▸), the penetration of the atomic ρ-basin of the electron occupier into the φ_es_-basin of the neighboring electron contributor was accurately defined as a manifestation of the *interatomic charge (electron) transfer* in the position space, resulting from the formation of any many-electron multinuclear system from free atoms, which was first introduced by Fayzullin and coworkers (Shteingolts, Stash *et al.*, 2021 ▸; Shteingolts *et al.*, 2022 ▸) and further developed by Kartashov *et al.* (2023 ▸) and Saifina *et al.* (2023 ▸). In other words, it describes the atomic or even sub­atomic contributions made by the immediate environment to the formation of the charge of the atom in question, driven by the electropositivity and electronegativity of the atoms involved (Kartashov *et al.*, 2023 ▸). In particular, the direction of the interatomic electron transfer is the opposite to that of the force **F**_es_(**r**) acting inside the gap between the φ_es_- and ρ-basins. This opens up the possibility of estimating the true, direction-dependent *electronegativity* of actual chemically bonded atoms (Shteingolts *et al.*, 2022 ▸; Saifina *et al.*, 2023 ▸): the more electronegative ∇ρ-atom, behaving as an electron occupier, penetrates the adjoining **F**_es_-pseudoatom, while the **F**_es_-pseudoatom of the more electropositive atom becomes penetrated, *i.e.* the latter acts as an electron contributor. Furthermore, the pseudoatomic φ_es_-basin thus serves as a kind of reference for tracking the change of the corresponding atomic ρ-basin due to the formation of a multinuclear system (Takebayashi *et al.*, 2023 ▸). We have recently applied the phenomena of ρ-to-φ_es_-basin interpenetration between a metal atom and atoms of the first coordination sphere to the real space description of the ligand-binding field for coordination compounds (Takebayashi *et al.*, 2023 ▸).

The nature of the gap between the φ_*k*_- and ρ-basins related to the neighboring nuclei is more challenging and implies the quantum chemical interaction picture between atoms (Shteingolts *et al.*, 2022 ▸; Saifina *et al.*, 2023 ▸). According to both experimental (Tsirelson & Stash, 2020 ▸; Shteingolts, Stash *et al.*, 2021 ▸; Stash *et al.*, 2021 ▸; Shteingolts, Voronina *et al.*, 2021 ▸; Shteingolts *et al.*, 2022 ▸; Kartashov *et al.*, 2023 ▸; Saifina *et al.*, 2023 ▸) and theoretical (Kartashov *et al.*, 2023 ▸; Saifina *et al.*, 2023 ▸) data, it was found that the local kinetic force **F**_*k*_(**r**) originating from the electron contributor interpenetrates across the ZFS *S*(Ω) for various polar interactions. The simultaneous partitioning of a many-electron multinuclear system in the real space into ∇ρ-atoms and **F**_es_- and **F**_*k*_-pseudoatoms was first introduced by Shteingolts, Stash *et al.* (2021 ▸) and helped attribute physical meaning to the gap between the φ_*k*_- and ρ-basins. First and foremost, the **F**_*k*_-pseudoatoms could be perceived as pseudoatoms, whose behavior is corrected for the electron exchange effect (Shteingolts *et al.*, 2022 ▸; Kartashov *et al.*, 2023 ▸). Second, in all cases known to date, the ZFS *P*(Ω) is observed somewhere between the ZFSs *U*(Ω) and *S*(Ω) when considering the internuclear region, thus dividing the zone of interatomic charge transfer into two parts (Shteingolts, Stash *et al.*, 2021 ▸; Shteingolts *et al.*, 2022 ▸). Third, for covalent bonds, the surface *P*(Ω) closely approaches the surface *U*(Ω) within the internuclear region, so that almost the entire or most of the volume of interatomic charge transfer is found to be the overlapping gap in question (Shteingolts, Voronina *et al.*, 2021 ▸; Shteingolts *et al.*, 2022 ▸). Fourth, the stronger an O⋯H hydrogen bond is, the deeper the hydrogen **F**_*k*_-pseudoatom usually intersects with the oxygen ∇ρ-atom (Shteingolts *et al.*, 2022 ▸; Kartashov *et al.*, 2023 ▸). Finally, there is a correlation between the φ_*k*_-to-ρ-basin penetration depth for polar noncovalent interactions and a decrease in the PAEM barrier and an increase in the kinetic potential well (Saifina *et al.*, 2023 ▸), which are, in turn, proposed as measures of covalency (Shteingolts *et al.*, 2022 ▸; Saifina *et al.*, 2023 ▸). All this together allowed Fayzullin and coworkers to assert that the gap between the φ_*k*_- and ρ-basins is the hitherto unknown quantum-chemical phenomenon in the position space, manifesting the *quantum-chemical response against the pure interatomic charge transfer* or, in other words, *transferred ED sharing* (Shteingolts *et al.*, 2022 ▸). At equilibrium, the ED belonging to the ∇ρ-atom falls into the **F**_*k*_-pseudoatom related to the neighboring nucleus and is consequently pulled toward this nucleus by the FAEM 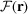 and pushed out of the same nucleus by the kinetic force **F**_*k*_(**r**), both originating from the **F**_*k*_-pseudoatom (Fig. 1 ▸), thus reflecting the covalence of a polar interatomic interaction (Shteingolts *et al.*, 2022 ▸). In other words, the ED of an atom appearing in the ρ-to-φ_*k*_-basin penetration gap is attracted to the nucleus of the neighboring atom by the electrostatic **F**_es_(**r**) total static 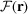 forces. Furthermore, the interatomic electron transfer and the accompanying sympathetic quantum-chemical response can be represented by the concepts of electronic effects in a molecule and of the crystal packing effect (Kartashov *et al.*, 2023 ▸).

The phenomena, regularities, observations and interpretations, described above and deliberately omitted for the sake of brevity, formed the basis of the currently developing orbital-free *quantum-topological binding approach* to the *mechanical* description of chemical structure and processes, which deals with the force density fields of kinetic and static nature (Shteingolts *et al.*, 2022 ▸; Saifina *et al.*, 2023 ▸). Herein, we have tried to show the great potential of the binding approach and its non-trivial focus on chemical and crystallographic problems. However, its application requires time-consuming theoretical calculations for many-electron systems (Kartashov *et al.*, 2023 ▸) and/or hard-to-obtain experimental EDs reconstructed from high-resolution diffraction data. Note that the components of equation (3) can be expressed in terms of the multipole-modeled experimental ED and its derivatives, using the orbital-free density functional theory approximations (Stash & Tsirelson, 2022 ▸). There is a long list of difficulties that an experimental crystallographer faces in obtaining accurate ED distributions (Tsirelson & Ozerov, 1996 ▸; Herbst-Irmer & Stalke, 2017 ▸; Shteingolts, Voronina *et al.*, 2021 ▸). Fortunately, the method based on the use of the transferable multipole pseudo-atoms allows one to obtain the semi-experimental semi-theoretical transferable aspherical pseudo-atom models (TAAMs) of crystal structures, being refined against the experimental structure factors of accessible resolution and accuracy (Brock *et al.*, 1991 ▸; Pichon-Pesme *et al.*, 1995 ▸; Volkov *et al.*, 2004 ▸; Dittrich *et al.*, 2004 ▸; Domagała *et al.*, 2012 ▸; Kumar *et al.*, 2019 ▸). Such models proved to be sufficient for obtaining EDs in the crystals of small molecules and proteins, as well as for calculating the electrostatic potential and the electrostatic interaction energy, all of which are comparable with experimental and purely theoretical data (Lecomte *et al.*, 2005 ▸; Zarychta *et al.*, 2007 ▸; Dominiak *et al.*, 2007 ▸; Bąk *et al.*, 2011 ▸). Based on the above, we have concluded on the timeliness and importance of testing the applicability of transferable multipole pseudo-atoms for restoring inner-crystal electronic force density fields, which, in turn, provide new insights into chemical structure and crystalline organization.

## 2. Experimental and computational methods

### 2.1. Multipole models

The single-crystal X-ray diffraction experiment for 1,3-bis­(2-hy­droxy­ethyl)-6-methyl­pyrimidine-2,4(1*H*,3*H*)-dione was performed on a Bruker D8 QUEST diffractometer equipped with a PHOTON III area detector and an IµS DIAMOND microfocus X-ray tube (Mo *K*α radiation, λ = 0.71073 Å), the diffractometer was equipped with an Oxford Cryostream LT instrument. Data were collected at 100 K according to the recommended strategy in a φ/ω-scan mode with a frame width of 0.5°. The data reduction package *APEX4* (Bruker, 2021 ▸) was used for data processing and correction. The structure was solved using *SHELXT* (Sheldrick, 2015 ▸). Charge-density refinement was performed within the multipole formalism of Hansen & Coppens (1978 ▸) as implemented in the *MoPro* software package (Jelsch *et al.*, 2005 ▸). Here, the total quasistatic ED ρ(**r**) is considered as a superposition of the EDs contributed by each pseudo-atom τ, expressed as the sum of the spherical core, spherical valence and deformation valence contributions. Each nucleus-centered pseudo-atomic ED ρ_τ_(**r** − **r**_0_) is expressed as the series expansion in real spherical harmonic functions *Y*_*lm*_ through the order *l*_max_:

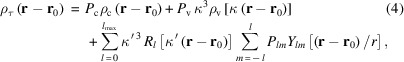

where **r**_0_ is the nuclear coordinates; *P*_c_, *P*_v_ and *P*_*lm*_ are the core, valence and multipole population coefficients, respectively; κ and κ′ are the spherical and deformation valence expansion/contraction parameters; and *R*_*l*_(**r** − **r**_0_) are the normalized exponential Slater-type radial functions. The core and spherical valence scattering factors from Su & Coppens (1997 ▸) were used. The radial functions *R*_*l*_ with parameters *n*_*l*_ = 2, 2, 3, 4 for oxygen, nitrogen and carbon pseudo-atoms, *n*_*l*_ = 1, 2 for hydrogen pseudo-atoms and the values of the orbital exponents ζ_O_ = 4.4974, ζ_N_ = 3.8106, ζ_C_ = 3.1303 and ζ_H_ = 2.0000 were used. Multipole refinement was performed against *F* with the reflections satisfying the *I* > 2σ(*I*) condition. A reciprocal resolution sin(θ_max_/λ) of the data was 1.38 Å^–1^. The unit cell electroneutrality constraint was imposed. The C—H and O—H bond distances were constrained and restrained to the theoretical values obtained from the optimized periodic structure (see below), respectively. The same deformation valence expansion/contraction parameter κ′ was used for all multipole levels of each pseudo-atom. The multipole expansion was truncated at the hexadecapolar level (*l*_max_ = 4) for the non-hydrogen pseudo-atoms; the multipole population *P*_00_ was set to zero. Reasonable local symmetry constraints were applied: *mm*2 for O4, 3 for C61 and *m* for the other pseudo-atoms. For each hydrogen pseudo-atom, the monopole population *P*_v_ and the bond-oriented dipole population *P*_10_ (C—H and O—H), as well as the bond-oriented quadrupole population *P*_20_ for H1 and H3 in O—H, were refined. Anisotropic displacement parameters were calculated for the hydrogen pseudo-atoms using the *SHADE3* algorithm and inserted several times between the refinement steps until no further change was achieved (Madsen, 2006 ▸).

The supplementary theoretical multipole model (TMM) with the optimized geometry (see below) was fitted to all calculated static structure factors truncated to sin(θ_max_/λ) = 1.39 Å^–1^: *R*(*F*) = 0.0067, *wR*(*F* ^2^) = 0.0130 and *S*(*F*) = 1.100.

The TAAM model was prepared using the *LSDB* code by transferring the relevant multipole parameters from the University at Buffalo Data Bank (UBDB2018) of aspherical pseudo-atoms obtained by Fourier space fitting to *ab initio* calculated molecular EDs (Volkov *et al.*, 2004 ▸; Kumar *et al.*, 2019 ▸). After the data transfer, the refinement of the remaining 178 parameters against the experimental structure factors was as follows: *R*(*F*) = 0.0196, *wR*(*F* ^2^) = 0.0326 and *S*(*F*) = 0.998.

#### 2.1.1. Crystallographic data summary

The crystallographic data for the experimental multipole model (EMM) are as follows: colorless prism (0.340 × 0.402 × 0.457 mm), melting point = 111°C; C_9_H_14_N_2_O_4_, *M*_r_ = 214.22 g mol^–1^; monoclinic space group *P*2_1_/*n* (No. 14), unit-cell parameters: *a* = 7.8840 (3) Å, *b* = 7.2121 (3) Å, *c* = 16.7362 (6) Å, β = 94.4437 (3)°, *V* = 948.76 (6) Å^3^; *Z* = 4, *Z*′ = 1, *F*(000) = 456, *D_x_* = 1.500 g cm^−3^ and μ = 0.119 mm^−1^; *T*_max_/*T*_min_ = 0.9253/0.8642; 580 996 reflections were collected (2.778° ≤ θ ≤ 78.721°, index ranges: −21 ≤ *h* ≤ 21, −19 ≤ *k* ≤ 19 and −44 ≤ *l* ≤ 41), of which 20 648 were unique (*R*_σ_ = 0.0107, *R*_int_ = 0.0357), sin(θ_max_/λ) = 1.380 Å^–1^, completeness to θ_max_ is 98.8%. The final refinement of 436 variables for 19 363 observed reflections with *I* > 2σ(*I*) converged to the following figures of merit: *R*(*F*) = 0.0182, *wR*(*F* ^2^) = 0.0286, *S*(*F*) = 1.005, (Δ/σ)_max_ = −0.007 and 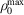 / 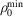 = +0.154/–0.312 e Å^–3^ (root-mean-square deviation = 0.052 e Å^–3^). Hereinafter, symmetry operation codes are indicated by the following superscripts: *a* = 1 – *x*, 1 – *y*, 1 – *z*; *b* = 1.5 – *x*, *y* + 0.5, 0.5 – *z*; *c* = *x*, *y* – 1, *z*, *d* = *x*, *y* + 1, *z*.

The details for the absorption correction, the experimental coordinates, the thermal parameters, the pseudo-atom parametrization, the pseudo-atom populations in the global frame and the molecular geometry information can be found in the CIF provided in the supporting information (CCDC 2259862).

### 2.2. Theoretical computations

The crystal structure was optimized with constant unit-cell parameters at the ω*B97X/pob-TZVP-rev2* level (Chai & Head-Gordon, 2008*b* ▸; Oliveira *et al.*, 2019 ▸) using the *CRYSTAL17* software. The experimental geometry was taken as the initial geometry. Truncation criteria values for bielectronic integrals were set as follows: the overlap threshold for Coulomb integrals and Hartree–Fock exchange integrals was set to 10^–8^ a.u.; the penetration threshold for Coulomb integrals was set to 10^–8^ a.u.; and the first and second criteria for pseudo-overlap were set to 10^–8^ and 10^–24^ a.u., respectively. The total energy convergence tolerance was set to 10^–10^ a.u. The shrinking factors of 8 for the Monkhorst net and 16 for the Gilat net were used, resulting in 170 points in the irreducible part of the Brillouin zone. All the vibrational frequencies computed at the Γ-point were positive.

The dimer formed by a pair of hydrogen bonds was isolated from the crystal structure and optimized at the ω*B97X-D* functional (Chai & Head-Gordon, 2008*a* ▸) and *aug-cc-pVTZ* basis sets (Dunning, 1989 ▸) using the *Gaussian16* software (Frisch *et al.*, 2016 ▸). No imaginary vibrational frequencies were found.

### 2.3. Analysis

Calculations and analyses within the framework of orbital-free quantum crystallography based on the static EDs reconstructed from the multipole models were carried out using the *WinXPRO*, *3DPlot* and *TrajPlot* software (Stash & Tsirelson, 2014 ▸, 2022 ▸). All procedures used have been reported previously (Shteingolts, Stash *et al.*, 2021 ▸; Kartashov *et al.*, 2023 ▸). The calculations were performed within a finite spherical electroneutral cluster with a radius of 14 Å. An out-of-plane distance of 0.8 Å was set up for the calculation of gradient maps. The electrostatic potential φ_es_(**r**) is accessible from the multipole-described ED (Su & Coppens, 1992 ▸). The exchange-correlation potential φ_*x*_(**r**) was approximated according to von Barth & Hedin (1972 ▸). The last two functions are also needed to obtain the PAEM 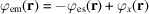 and the relative kinetic potential 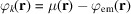 , where μ(**r**) is the electronic chemical potential (see below). Some other formulae used can be found in the *Introduction* [Sec sec1] and the *Results and discussion* [Sec sec3]. In this work, we calculated μ for the crystal to be −0.1578 a.u., as the negative half-sum of the inverse energies of the lowest unoccupied and highest occupied orbitals (Parr & Yang, 1989 ▸; Kartashov *et al.*, 2023 ▸).

The hydrogen bond energies (in kcal mol^–1^) were estimated by the widely used correlations 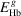 = 269.2014*g_b_* and 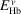 = −313.7545*v_b_* (Espinosa *et al.*, 1998 ▸; Mata *et al.*, 2011 ▸; Vener *et al.*, 2012 ▸), where *g_b_* and *v_b_* are the kinetic and potential energy densities at a BCP (in a.u.), respectively. For the multipole-derived EDs, the approximation by Kirzhnits (1957 ▸) was used in conjunction with the local form of the virial theorem to obtain *g_b_* and *v_b_*.

The topological analysis of the periodic ED was performed using *TOPOND*14 (Gatti *et al.*, 1994 ▸).

The wavefunction obtained for the gas-phase optimized hydrogen-bonded dimer was analyzed by means of the *Multiwfn 3.8*(*dev*) software (Lu & Chen, 2012 ▸). In this case, the built-in code by Zhang & Lu (2021 ▸) and the formula by Müller (1984 ▸) were used to evaluate φ_es_(**r**) and φ_*x*_(**r**), respectively. For the dimer, μ = −0.1442 a.u., as the negative half-sum of the inverse energies of the lowest unoccupied and highest occupied orbitals. A detailed procedure for the generation of theoretical trajectory maps is described by Kartashov *et al.* (2023 ▸).

## 3. Results and discussion

In this work, the inner-crystal electronic, potential and force-field structure of 1,3-bis­(2-hy­droxy­ethyl)-6-methyl­pyrimidine-2,4(1*H*,3*H*)-dione has been studied by high-resolution single-crystal X-ray diffraction at 100 K within the space-distributed multipole formalism of Hansen & Coppens (1978 ▸). There are some examples of experimental ED studies on the crystals of other uracil derivatives in the recent literature (Klooster *et al.*, 1992 ▸; Jarzembska *et al.*, 2012 ▸, 2017 ▸; Shteingolts, Saifina *et al.*, 2021 ▸; Shteingolts, Stash *et al.*, 2021 ▸). The crystal structure was also optimized using the Kohn–Sham method with periodic boundary conditions (Dovesi *et al.*, 2018 ▸) at the ω*B97X/pob-TZVP-rev2* level (Chai & Head-Gordon, 2008*b* ▸; Oliveira *et al.*, 2019 ▸). In addition, we prepared the aspherical pseudo-atom models with the parameters fitted to the theoretical static structure factors or taken from the University at Buffalo Data Bank (UBDB2018) (Volkov *et al.*, 2004 ▸; Kumar *et al.*, 2019 ▸). The main objective of this work was to verify the applicability of transferable aspherical pseudo-atoms for restoring the electronic force density fields **F**_es_(**r**), **F**_*k*_(**r**) and 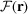 , as well as the fermionic potential φ_f_(**r**), in crystals within covalent bonds and classical and nonclassical hydrogen interactions, exemplified by the aforementioned 6-methyl­uracil derivative. The results were also supported by the analysis of a gas-phase optimized hydrogen-bonded dimer exhibiting a uracil–uracil π-stacking interaction.

For convenience, in this paper, we distinguish between the terms pseudoatom and pseudo-atom with different spellings: The former was introduced by Fayzullin and coworkers (Shteingolts *et al.*, 2022 ▸; Kartashov *et al.*, 2023 ▸) and is defined by equations (2) or (3), whereas the latter was introduced by Stewart (1976 ▸) and is described by equation (4).

The importance of various types of hydrogen bonds in chemical crystallography, crystal engineering and molecular biology is so great that it needs no further justification. Suffice it to note their prevalence in the crystals of organic compounds, their preferred directionality and the large variability in dissociation energy *E*_Hb_. The latter can be attributed to the varying degrees of covalency (Grabowski, 2011 ▸) or, in other words, to the sharing of the ED and the transferred charge, among the differently arranged inter­actions (Shteingolts *et al.*, 2022 ▸; Saifina *et al.*, 2023 ▸).

### 3.1. Molecular and crystal structure

**Figure 2:**
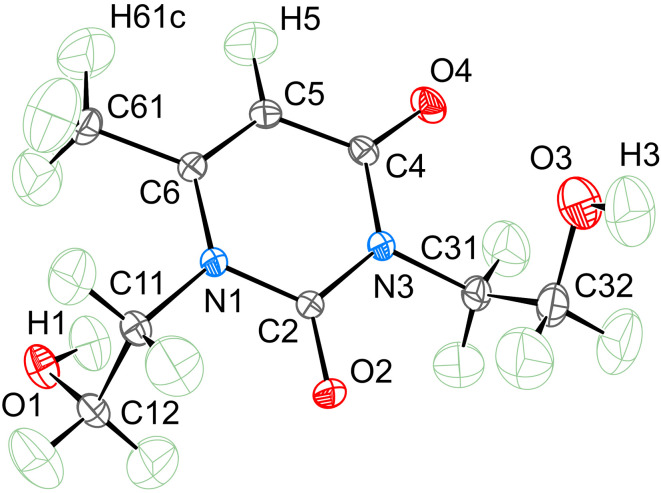
Molecular geometry of 1,3-bis­(2-hy­droxy­ethyl)-6-methyl­uracil in the crystal structure according to the high-resolution single-crystal X-ray diffraction data at 100 K. Anisotropic displacement ellipsoids are shown at the 80% probability level. The partial atomic numbering scheme adopted in this article is given.

According to the single-crystal X-ray diffraction experiment performed, the uracil derivative crystallizes in the monoclinic space group *P*2_1_/*n* and is represented by a single molecule per the asymmetric cell. The geometry of the molecule in the crystals is shown in Fig. 2 ▸. It carries acceptors and donors of hydrogen interactions, such as carbonyl and hydroxyl groups, so it is not surprising that the main crystal-forming motif in the crystals is built up by classical hydrogen bonds of the O—H⋯O[=C] type.

**Figure 3:**
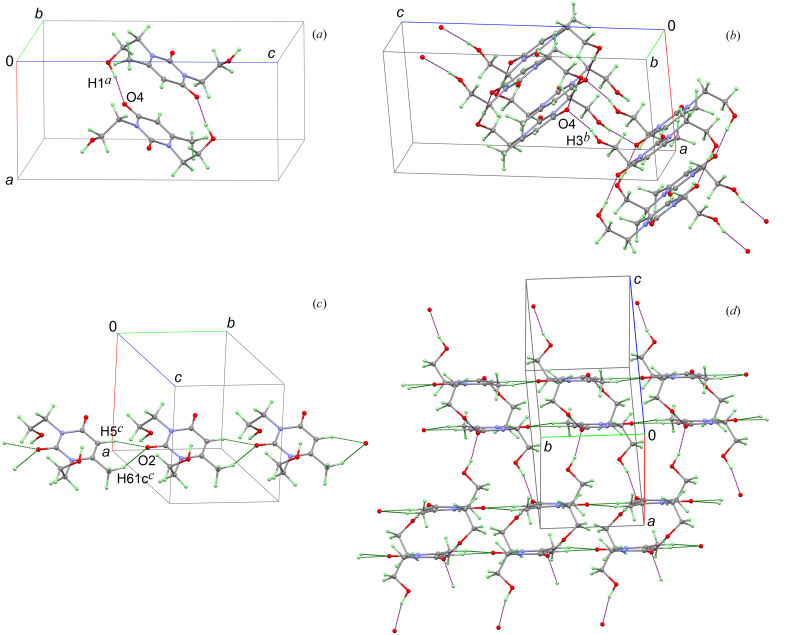
Fragments of the molecular packing of 1,3-bis­(2-hy­droxy­ethyl)-6-methyl­uracil in the crystal according to the diffraction data: (*a*) centrosymmetric hydrogen-bonded dimer, (*b*) and (*d*) two different views of the same hydrogen-bonded layer, and (*c*) selected chain formed by C—H⋯O[=C] interactions. Classical and nonclassical hydrogen bonds are shown as purple and green dashed lines, respectively.

A pair of symmetrically related intermolecular hydrogen bonds between the hydroxyl H1 hydrogen atoms and the carbonyl O4 oxygen atoms leads to the association of the two molecules to form a centrosymmetric dimer [Fig. 3 ▸(*a*)]. These dimers are further cross-linked into a two-dimensional structure (or layer), shown in Fig. 3 ▸(*b*), by the other set of equivalent intermolecular hydrogen bonds with the same O4 oxygen atoms but already with the hydroxyl H3 hydrogen atoms along the twofold screw axis. At the same time, each carbonyl O2 oxygen atom participates in two nonclassical hydrogen bonds with atoms H5[—C(*sp* ^2^)] and H61c[—C(*sp* ^3^)], which are approximately in the plane of the uracil heterocyclic fragment [Fig. 3 ▸(*c*)]. They also contribute to the maintenance of the layered structure [Fig. 3 ▸(*d*)]. The geometric parameters of the aforementioned hydrogen bonds are listed in Table 1 ▸. The table also allows a comparison of the data obtained from the experimental multipole model with those obtained from the aspherical pseudo-atom model with parameters taken from the database, as well as from the optimized crystal structure. It can be seen that the geometric parameters of the experiment and the calculations are in good agreement. Because of various noncovalent interactions, mainly nonclassical hydrogen bonds, the layers are joined together to form a very dense crystal structure with a packing index of 78.9%.

### 3.2. Revealing noncovalent interactions

The analysis of the ED ρ(**r**) and the physically grounded functions derived from it makes it possible to describe the structure of a system at the subatomic level. The quantum-topological analysis (Bader, 1990 ▸, 1991 ▸) of the experimental and theoretical ED reveals the BCP and the accompanying bond path between the respective hydrogen and oxygen atoms for each of the four hydrogen bonds discussed. Within the QTAIM, the bond path is postulated to be an indicator of chemical bonding. The selected characteristics of the BCPs are listed in Table 2 ▸. Note that there is a good agreement between the data for different models, including those calculated from the wavefunction for the optimized crystal structure. The following general trend can be seen in Tables 1 ▸ and 2 ▸: the shorter the H⋯O and O⋯O interatomic distances *d*_H⋯A_ and *d*_D⋯A_ and the larger the angle 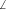 DHA (or 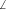 O—H⋯O), the higher the values of the ED at the BCP ρ_b_ and the approximate interaction energy *E*_Hb_ are observed. The interaction [O1*^a^*—]H1*^a^* ⋯O4 forming the centrosymmetric dimers [Fig. 3 ▸(*a*)] is the strongest noncovalent interaction in the crystal and is significantly stronger than its counterpart [O3*^b^*—]H3*^b^* ⋯O4 (Table 2 ▸).

Let us consider another approach to detecting interatomic interactions. Atomic basins or ED isosurfaces with the electrostatic potential φ_es_(**r**) mapped on them are widely used to describe noncovalent interactions in associates, coordination compounds, protein–ligand complexes and crystals. However, the electronic exchange effect, which is ignored in this approach, plays the ultimate role, especially within inter­atomic regions. The consideration of the exchange effect in the construction of such heat-mapped surfaces seems to be particularly important for supramolecular-synthon-forming interactions, such as π⋯π, LP⋯π, hydrogen, halogen and chalcogen bonds, as well as for coordination bonds. This issue has recently been addressed, namely, we have proposed to represent the total static potential 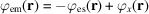 (Shteingolts, Stash *et al.*, 2021 ▸; Kartashov *et al.*, 2023 ▸) and the magnitude of the associated total static force 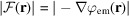 (Kartashov *et al.*, 2023 ▸) on atomic basins or ZFSs *S*(Ω). Such maps can be interpreted in several ways: more negative values of the PAEM 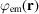 correspond to easier electron sharing between atoms through the interatomic boundary *S*(Ω), and when considering the internuclear region, the FAEM 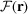 crosses this boundary and is directed toward the electron contributor (*e.g.* a hydrogen atom in an H⋯O interaction). Higher values of 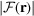 at the boundary surface imply a stronger action of 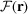 and **F**_*k*_(**r**) through the ZFS *S*(Ω), which, among other things, indicates a deeper ρ-to-φ_*k*_-basin penetration and thus a larger sharing of the transferred ED. Importantly, the observation of high 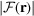 at the atomic surfaces is typical for polar interactions, whereas relatively low PAEM values are expected for any chemical bond. Due to 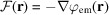 , the PAEM mapped on the surface allows the tracking of the FAEM vector thereon, whereby the force acts in the direction of decreasing the PAEM value. Hence, the proposed approach makes it possible to *probe* the surface of a molecule in a crystal or associate and obtain relevant and, as far as possible, complete information about the surface state and chemical environment. To generalize, a local decrease in the PAEM values plotted at the interatomic boundary is expected for any bonding interatomic interaction, in contrast to the FAEM values plotted at the same boundary, which range from close to zero for nonpolar values to larger or even maximum values for polar bonds. We highly recommend it as a replacement for similar but ill-conditioned φ_es_(**r**)-mapped interatomic surfaces. We expect our approach to provide robust structural results for protein–ligand complexes, including the models based on the TAAM.

**Figure 4:**
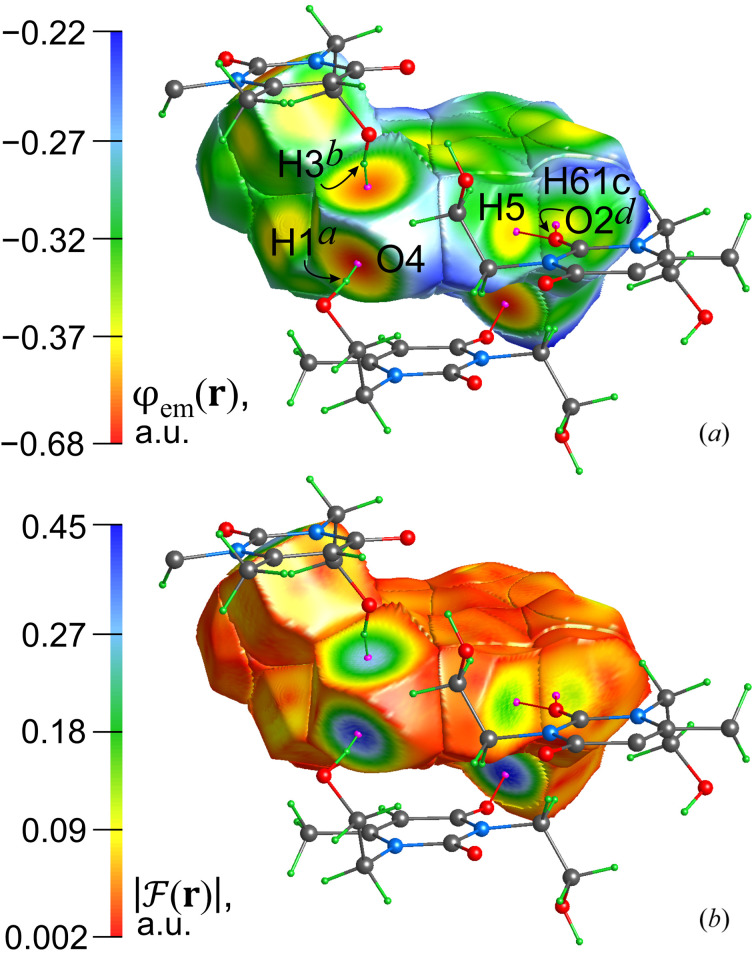
Heat maps of (*a*) the potential acting on an electron in a molecule φ_em_(**r**) and (*b*) the magnitude of the force acting on an electron in a molecule 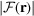 , distributed over the inner-crystal atomic ρ-basins of 1,3-bis­(2-hy­droxy­ethyl)-6-methyl­uracil. BCPs for the hydrogen bonds and the associated bond paths are shown as magenta spheres and thin lines. Three neighboring molecules of the cluster are also shown. The data are derived from the experimental charge density.

Fig. 4 ▸ shows a selected cluster of four molecules isolated from the crystal structure, with the central one represented by its inner-crystal atomic basins. On the surface *S*(Ω), the values of the PAEM function [Fig. 4 ▸(*a*)] or the FAEM magnitude [Fig. 4 ▸(*b*)] derived from the EMM have been heat mapped. It is clearly seen that the concentric areas of reduced potential and increased force correspond to the discussed polar interactions. Furthermore, this picture is more pronounced where the interaction is stronger (Table 2 ▸). Thus, such heat maps allow us not only to identify interactions but also to evaluate their hierarchy in the bonding structure of the crystal. The TAAM gives very similar results and could therefore be considered to be used to reveal and categorize noncovalent interactions.

### 3.3. Revealing electron lone pairs

One of the most commonly used ways to formally describe noncovalent interactions arranged according to the donor–acceptor or, in other words, Lewis mechanism, is to identify an LP donor or a Lewis base and an LP acceptor or a Lewis acid. In our previous works (Shteingolts & Fayzullin, 2020 ▸; Shteingolts, Saifina *et al.*, 2021 ▸; Shteingolts, Stash *et al.*, 2021 ▸; Karimata *et al.*, 2022 ▸; Takebayashi *et al.*, 2023 ▸), we have repeatedly used this approach, although we have also raised some important caveats regarding its applicability (Saifina *et al.*, 2023 ▸). Some caveats are also provided in this paper. The terms above should not be confused with the terms *contributor* and *occupier* (Shteingolts, Stash *et al.*, 2021 ▸).

**Figure 5:**
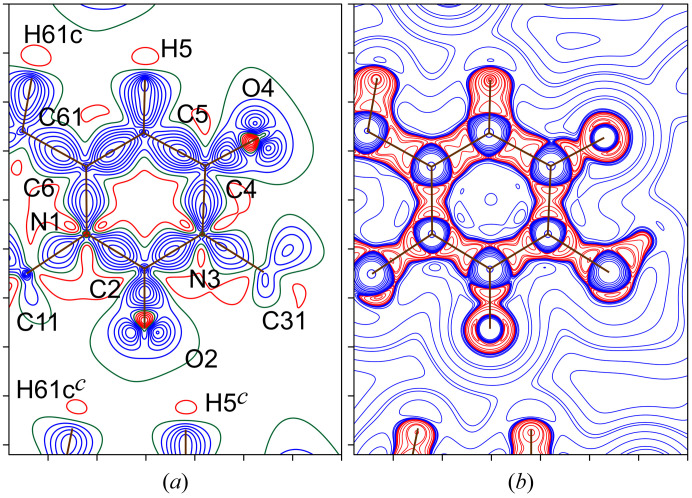
Contour maps of (*a*) the static deformation ED δρ(**r**) and (*b*) the Laplacian of the ED ∇^2^ρ(**r**) for 1,3-bis­(2-hy­droxy­ethyl)-6-methyl­uracil, calculated from the experimental multipole data. Blue, green and red isocontours correspond to positive, zero and negative function values, respectively. Contour steps are 0.1 e Å^–3^ for the δρ(**r**)-map; the logarithmic scale in the form ±1, 2, 4, 8 × 10*^n^* (−3 ≤ *n* ≤ 2) e Å^–5^ is adopted for the ∇^2^ρ(**r**)-map. The distance between the axis tick marks is 1 Å. Both maps are plotted on the same atomic plane of N1, N3 and C6.

The total ED maps are usually unsuitable for illustrating the subtle details of chemical bonding and subatomic arrangement. For this purpose, let us consider the distribution of the static deformation ED δρ(**r**) (Roux *et al.*, 1956 ▸), which measures the change in the ED at any point **r** as a result of the relaxation (deformation) of the non-existent modeled electron–nuclear system composed of spherical pseudo-atoms toward a relative actual system at the constant nuclear configuration. Thus, the distribution of δρ(**r**) reflects the ED rearrangement because of interatomic bonding; however, it requires a promolecular or procrystal reference. In our case, the map in Fig. 5 ▸(*a*) shows the expected ED accumulations (positive deformation) in regions along the covalent chemical bonds. At the same time, both fragments C2=O2 and C4=O4 exhibit the characteristic distribution of carbonyl groups, namely, a compact zone of negative deformation (δρ(**r**) < 0) on the bond line close to the oxygen nucleus and two lobes of positive deformation (δρ(**r**) > 0) localized on the sides of the bond in the non-binding region, similar to previous works (Ahmed *et al.*, 2013 ▸; Shteingolts, Stash *et al.*, 2021 ▸; Shteingolts, Voronina *et al.*, 2021 ▸). There are also electron reduction regions (‘vacancies’) behind the hydrogen nuclei H5 and H61c on the continuation of the internuclear lines, each of which is directed to some extent to the nearest O2 lobe of the neighboring molecule.

Another common way to describe the electronic structure of a system is to analyze the Laplacian of the ED ∇^2^ρ(**r**), which shows the ED curvature. Consideration of ∇^2^ρ(**r**) allows one to evaluate the electron concentration (∇^2^ρ(**r**) < 0) and depletion (∇^2^ρ(**r**) > 0) of the ED in a molecule without having to refer to a hypothetical reference state, such as a promolecule (Bader & Essén, 1984 ▸). Importantly, within the regions of space with ∇^2^ρ(**r**) < 0, the potential energy contribution dominates over the kinetic energy in the local total electronic energy, although the converse is not always true. For the uracil derivative [Fig. 5 ▸(*b*)], the aforementioned ED accumulation peaks at the oxygen atoms O2 and O4 outside the carbonyl bonds are accompanied by significant ED concentrations expressed by the minimum CPs (3, +3) of ∇^2^ρ(**r**). These elements are called the valence shell charge concentrations (VSCCs) and, if applicable, they are usually identified with the electron LPs; for example, a recent example of this can be found in the work by Takebayashi *et al.* (2023 ▸). It can be seen that the bonding and nonbonding VSCCs located by ∇^2^ρ(**r**) match the regions of the ED accumulation [Fig. 5 ▸(*b*)].

In practice, the ED distribution is virtually incapable of indicating the location of LPs or π-density attributed to Lewis bases. Aside from the Laplacian of the ED ∇^2^ρ(**r**), there are actually a few robust and physically grounded functions that allow us to indicate an LP. For crystals, some of these can be evaluated from the experimental ED, albeit using the kinetic energy density approximation (Kirzhnits, 1957 ▸; Astakhov *et al.*, 2016 ▸). These include the local electronic temperature (Ghosh *et al.*, 1984 ▸; Shteingolts, Voronina *et al.*, 2021 ▸), the electron localization function (ELF) (Becke & Edgecombe, 1990 ▸; Savin *et al.*, 1991 ▸; Tsirelson & Stash, 2002*b* ▸), the localized orbital locator (LOL) (Schmider & Becke, 2000 ▸, 2002 ▸; Tsirelson & Stash, 2002*a* ▸), the phase-space-defined Fisher information density (PS-FID) (Astakhov & Tsirelson, 2014 ▸) *etc*. In our previous works, we promoted the use of one-electron potentials appearing in the Euler–Lagrange equation for ED to describe chemical bonding (Shteingolts, Stash *et al.*, 2021 ▸; Shteingolts *et al.*, 2022 ▸; Kartashov *et al.*, 2023 ▸; Saifina *et al.*, 2023 ▸). The distributions of three of them, namely, the von Weizsäcker (bosonic) potential φ_W_(**r**) (Herring, 1986 ▸; Hunter, 1986 ▸), the Pauli potential φ_P_(**r**) (March, 1986 ▸) and the fermionic potential φ_f_(**r**) (Tsirelson & Stash, 2021 ▸) indicate the electronic structure of the electron–nuclear system and reveal the spatial localization of LPs and π-density (Shteingolts, Stash *et al.*, 2021 ▸; Shteingolts *et al.*, 2022 ▸; Kartashov *et al.*, 2023 ▸; Saifina *et al.*, 2023 ▸). Moreover, the first two are determined by kinetic factors and form the total kinetic potential φ_*k*_(**r**), while the latter takes into account both kinetic and static quantum effects. The exact expression for φ_W_(**r**) for closed-shell systems is as follows:

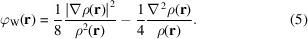

This function can distinguish the classically allowed regions and the ED concentration (φ_W_(**r**) > 0) from the forbidden regions for electrons and the ED depletion (φ_W_(**r**) < 0). At the same time, the fermionic potential φ_f_(**r**) is defined as the sum of the Pauli and exchange potentials:

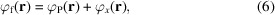

and is able to identify the repulsive [φ_f_(**r**) > 0] or attractive [φ_f_(**r**) < 0] local fermionic contribution to the electronic energy; thus, φ_f_(**r**) < 0 indicates regions where the static exchange correlation dominates over the kinetic exchange correlation. In lieu of equation (6), according to the Euler–Lagrange equation for ED, one can calculate φ_f_(**r**) using the following expression:

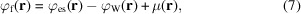

applying which significantly reduces the computational cost and, more importantly, does not require any approximation of φ_*x*_(**r**) nor does it depend on its choice (Saifina *et al.*, 2023 ▸). The electrostatic potential φ_es_(**r**) is available from the multipole-described ED (Su & Coppens, 1992 ▸). Here, μ(**r**) is the electronic chemical potential, which is equal to a negative near-zero constant μ for an equilibrium system. For simplicity, it can be set equal to zero (Tsirelson *et al.*, 2013 ▸), keeping this rough approximation in mind later in the analysis. It can also be estimated using the empirical formula proposed by Tsirelson & Stash (2021 ▸); unfortunately, μ calculated in this way is usually very underestimated. In this work, we calculated μ for the crystal to be −0.1578 a.u., as the negative half-sum of the inverse energies of the lowest unoccupied and highest occupied orbitals (Parr & Yang, 1989 ▸; Kartashov *et al.*, 2023 ▸).

**Figure 6:**
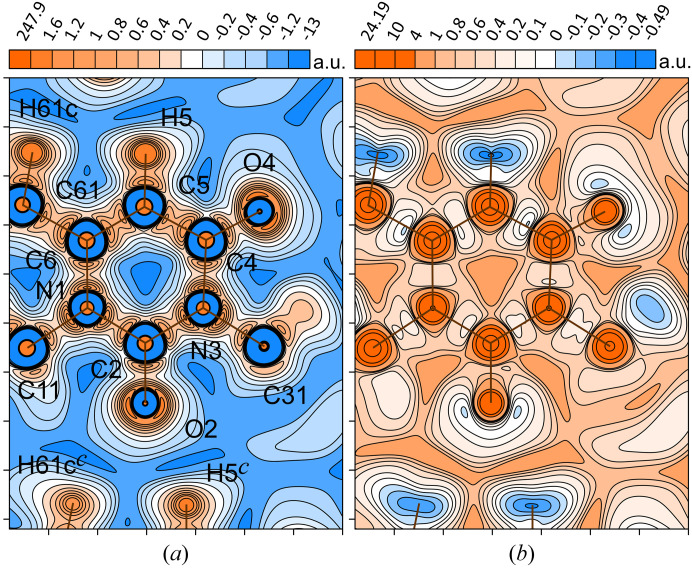
Contour maps of the inner-crystal (*a*) bosonic and (*b*) fermionic potentials, φ_W_(**r**) and φ_f_(**r**), for 1,3-bis­(2-hy­droxy­ethyl)-6-methyl­uracil, calculated from the experimental multipole-derived ED. Contour intervals of 0.2 a.u. are used; additional contours are shown at −1.2 and 1.6 a.u. for φ_W_(**r**); and −0.3, ±0.1, 4 and 10 a.u. for φ_f_(**r**). Function scale bars are presented above the maps. The distance between adjacent axis tick marks is 1 Å. Both maps are plotted on the same atomic plane of N1, N3 and C6.

Fig. 6 ▸ shows the contour maps of the distributions of the inner-crystal bosonic potential φ_W_(**r**) [Fig. 6 ▸(*a*)] and the inner-crystal fermionic potential φ_f_(**r**) [Fig. 6 ▸(*b*)], calculated on the basis of the experimental multipole-derived ED in the same map plane as in Fig. 5 ▸. Bonding and nonbonding VSCCs satisfy the condition φ_W_(**r**) > 0, whereas covalent bonds, π-density and LPs are expected to be found in the regions of negative and near-zero values of φ_f_(**r**). Here, we associate the position of LP with compact domains including the local maximum in φ_W_(**r**) or the local minimum in φ_f_(**r**). Thus, based on the experimental data, we have come to a rather obvious conclusion: each carbonyl oxygen atom, O2 and O4, carries two LPs aligned with the plane of the uracil-derivative heterocycle. At the same time, the oxygen atom O2, acting as a Lewis base, provides its LPs to the hydrogen atoms H5*^c^* and H61c*^c^*, acting as Lewis acids, to form the hydrogen bonds [C5*^c^*—]H5*^c^* ⋯O2 and [C61*^c^*—]H61c*^c^* ⋯O2. Interestingly, the φ_f_(**r**)-distribution distinguishes amide and urea functional groups with a deeper potential well along the C4—C5 covalent bond of the former than that for N1—C2, N3—C2 or N3—C4 [Fig. 5 ▸(*b*)]. The regions near the hydrogen nuclei, which are sparsely populated with electrons, exhibit deep potential wells with φ_f_(**r**) < 0. Furthermore, similar maps have been constructed for the aspherical pseudo-atom model with parameters adopted from the database (Fig. S1 of the supporting information). They show similar features to the experimental ones. Now we can state that the model built from transferable pseudo-atoms can reproduce the expected behavior of φ_W_(**r**) and φ_f_(**r**) comparable to the experimental results.

Comparing the maps in Figs. 5 ▸(*b*) and 6 ▸ may give the false impression that φ_W_(**r**) and φ_f_(**r**) show the same features of the subatomic structure as ∇^2^ρ(**r**). This is not the case. The fermionic potential φ_f_(**r**) makes it possible to distinguish an electron pair (or analog) involved in a noncovalent interaction from an uninvolved LP (Kartashov *et al.*, 2023 ▸). This is also demonstrated in this paper (*vide infra*). In addition, both potentials φ_W_(**r**) and φ_f_(**r**), complementing each other, reveal the arrangement of depleted subatomic regions (*e.g.* in metal atoms) that is hidden in the distribution of ∇^2^ρ(**r**) (Shteingolts *et al.*, 2022 ▸).

The preferred directionality of hydrogen bonds toward one of the two LPs of a carbonyl oxygen atom is known to be realized in crystals (Murray-Rust & Glusker, 1984 ▸; Olovsson, 1982 ▸; Taylor & Kennard, 1984 ▸; Wood *et al.*, 2008 ▸; Ahmed *et al.*, 2013 ▸). Most classical hydrogen bonds H⋯O[=C*X*_2_] are characterized by angles α_Hb_ 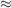 60° ± 10° and β_Hb_ < 15° (Ahmed *et al.*, 2013 ▸). Here, α_Hb_ is the angle between the straight line passing through the C=O internuclear line and the segment connecting the O atom and the projection of the H atom onto the plane of the carbonyl group *X*_2_C=O, and β_Hb_ is the angle between the *X*_2_C=O plane and the H⋯O internuclear line. In our case, the nonclassical hydrogen bonds [C5*^c^*—]H5*^c^* ⋯O2 and [C61*^c^*–]H61c*^c^* ⋯O2 show the β_Hb_ angles close to the ideal values, but the significantly reduced α_Hb_ angles. At the same time, the classical hydrogen bonds [O1*^a^*—]H1*^a^* ⋯O4 and [O3*^b^*—]H3*^b^* ⋯O4 occur far beyond the range of favorable or even acceptable directionality parameters α_Hb_ and β_Hb_, as shown in Table 1 ▸.

**Figure 7:**
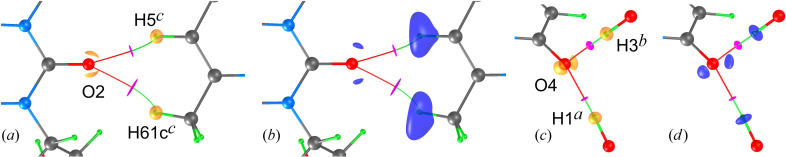
Isosurfaces of [(*a*) and (*c*)] the von Weizsäcker potential φ_W_(**r**) at 1.65 a.u. (orange) and [(*b*) and (*d*)] the fermionic potential φ_f_(**r**) at 0 a.u. (blue) in the crystal of 1,3-bis­(2-hy­droxy­ethyl)-6-methyl­uracil around the labeled atoms, derived from the experimental charge density. BCPs for the hydrogen bonds and the associated bond paths are shown as magenta discoid ellipsoids (Bohórquez *et al.*, 2011 ▸) and thin lines.

The isosurfaces of the von Weizsäcker potential φ_W_(**r**) and the fermionic potential φ_f_(**r**), shown in Fig. 7 ▸, reveal that the two classical hydrogen bonds [O1*^a^*—]H1*^a^* ⋯O4 and [O3*^b^*—]H3*^b^* ⋯O4 in the uracil derivative crystals are formed by a formally different mechanism that can hardly be attributed to the classical Lewis or donor–acceptor type. Indeed, in each case, the bond path or internuclear line does not cross or even pass close to the lobe of enhanced φ_W_(**r**) or negative φ_f_(**r**) potentials, associated with the location of the LP. Thus, it can be doubted that the O4 oxygen atom acts as a true Lewis base in the classical hydrogen bonds mentioned above, since geometrically and in actual fact, none of the LPs of the O4 atom are located within the binding region of the respective interaction. However, it is worth noting that there are still rather high φ_W_(**r**) values of less than 1.34–1.44 a.u. and low φ_f_(**r**) values exceeding 0.18–0.23 a.u. (the values are calculated based on experimental multipole data) in the vicinity of the carbonyl O4 oxygen atoms along the respective bond paths. Note that the observed behavior is by no means unique. For example, analogous hydrogen interactions can be found in urea (NH_2_)_2_C=O (Tsirelson & Stash, 2002*b* ▸) and the monoclinic racemic molecular compounds 1-benzyl-3-bromo-5-hy­droxy-4-[(4-methyl­phenyl)­sulfanyl]-1,5-di­hydro-2*H*-pyrrol-2-one (Gerasimova *et al.*, 2021 ▸) and 1-benzyl-3-bromo-4-[(4-chloro­phenyl)­sulfanyl]-5-hy­droxy-1,5-di­hydro-2*H*-pyrrol-2-one (Gerasimova *et al.*, 2022 ▸), which we have recently investigated.

### 3.4. Binding and interatomic charge transfer

**Figure 8:**
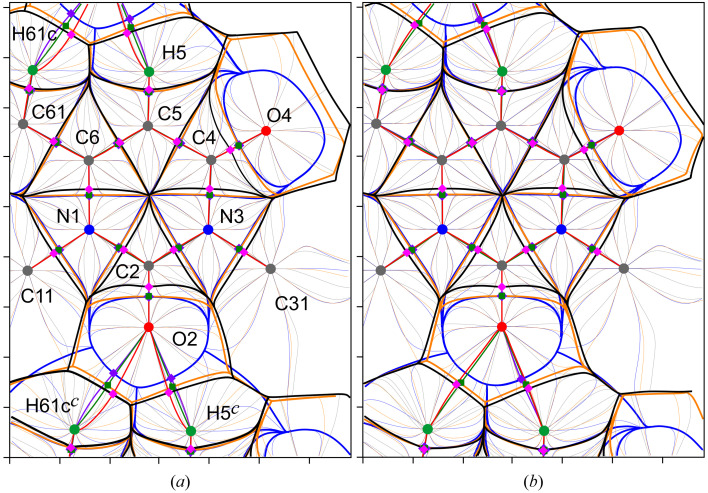
Superpositions of the (*a*) experimental or (*b*) semi-theoretical inner-crystal gradient fields of the ED ∇ρ(**r**) (black), the electrostatic potential ∇φ_es_(**r**) (blue) and the kinetic potential ∇φ_*k*_(**r**) (orange) for the uracil core of 1,3-bis­(2-hy­droxy­ethyl)-6-methyl­pyrimidine-2,4(1*H*,3*H*)-dione. The compared maps are calculated based on (*a*) the experimental multipole model (EMM) or (*b*) the aspherical pseudo-atom model with parameters taken from the database (TAAM). (Pseudo)atomic boundaries are highlighted with thicker lines of the corresponding color. Saddle CPs (3, −1) in ρ(**r**), φ_es_(**r**) and φ_*k*_(**r**) are indicated by magenta and violet rhombuses and green squares, respectively; the maximum CPs (3, −3) are shown as element-type-colored circles. Gradient paths connecting these CPs are colored red, violet and green, respectively. An out-of-plane distance is set to 0.3 Å. The distance between axis tick marks is 1 Å. The trajectory maps are plotted on the same atomic plane of N1, N3 and C6.

Let us now proceed to consider the behavior of the inner-crystal force density fields **F**_es_(**r**) and **F**_*k*_(**r**) and the characteristics of the **F**_es_(**r**)- and **F**_*k*_-pseudoatoms (or φ_es_- and φ_*k*_-basins) generated by these forces in the uracil derivative crystal. For this purpose, it was proposed here to use the superposition of trajectory maps with color-marked CPs found in the scalar fields ρ(**r**), φ_es_(**r**) and φ_*k*_(**r**), with thick black, blue and orange lines corresponding to the ZFSs *S*(Ω), *U*(Ω) and *P*(Ω), and with thin trajectories painted in the same colors, which are gradient lines, including the force ones. Figs. 8 ▸ and 9 ▸ show the superpositions of the trajectory maps of inner-crystal ∇ρ(**r**), **F**_es_(**r**) and **F**_*k*_(**r**) in the plane of the uracil derivative molecule and of the classical hydrogen bonds; Fig. S2 represents the (pseudo)atomic arrangement in the molecule plane according to the model with the parameters fitted to the theoretical static structure factors (TMM). When superimposed together, the three types of maps make it possible to visualize the gaps discussed and defined in the first part of the article (Shteingolts, Stash *et al.*, 2021 ▸). Recall that the realization of these gaps is one of the mechanisms by which atoms that have unequally exchanged the charge are held together (Shteingolts *et al.*, 2022 ▸; Saifina *et al.*, 2023 ▸). The atomic and pseudoatomic charges are listed in Table 3 ▸. The pseudoatomic charge *q*_**F**_*k*__ was calculated as the difference between the electron populations of a free atom of a chemical element and a corresponding **F**_*k*_-pseudoatom confined by a ZFS *P*(Ω); note that the **F**_es_-pseudoatoms are neutral.

The analysis of these maps complemented by the overlay of the three-dimensional basins, as well as the tabulated data, reveals that the pseudoatomic φ_es_- and φ_*k*_-basins of C2 extend and permeate into the atomic ρ-basins of the immediate surroundings, thus contributing a portion of the ED observed within the ρ-to-φ_es_-basin overlapping gaps. It can be concluded that the atom C2 (5.21 Å^3^ for EMM, 5.49 Å^3^ for TAAM and 5.32 Å^3^ for TMM) is highly positively charged (Table 3 ▸) and acts as an electron contributor to almost all of its surroundings. At the same time, the **F**_*k*_-pseudoatom C2 is much less charged, only slightly smaller in volume, compared with the **F**_es_-pseudoatom C2 (9.52 versus 10.61 Å^3^ for EMM, 9.92 versus 12.19 Å^3^ for TAAM, 9.81 versus 13.10 Å^3^ for TMM), and tends to take the shape of the **F**_es_-pseudoatom in the directions of the covalent bonds. About 79.4–84.0% of the transferred ED can be considered as shared within the neighboring occupier atoms, that is, presented within the ρ-to-φ_*k*_-basin penetration gaps.

The situation is reversed for the nitro­gen atoms N1 and N3 (Fig. 8 ▸). Their ρ-basins invade the neighboring pseudoatomic φ_es_- and φ_*k*_-basins, thus occupying a portion of the ED that is kept within the ρ-to-φ_es_-basin penetration gaps. The atoms N1 (10.54 Å^3^ for EMM, 10.48 Å^3^ for TAAM and 10.31 Å^3^ for TMM) and N3 (10.66 Å^3^ for EMM, 10.44 Å^3^ for TAAM and 10.29 Å^3^ for TMM) are highly negatively charged (Table 3 ▸) and behave as electron occupiers to their surroundings. The **F**_*k*_-pseudoatoms N1 and N3 are slightly larger in volume than the **F**_es_-pseudoatoms N1 (8.06 versus 6.22 Å^3^ for EMM, 8.12 versus 6.57 Å^3^ for TAAM, 8.08 versus 6.42 Å^3^ for TMM) and N3 (7.91 versus 6.01 Å^3^ for EMM, 8.09 versus 6.57 Å^3^ for TAAM, 8.07 versus 6.51 Å^3^ for TMM). However, they are again significantly less charged than the corresponding ∇ρ-atoms and again tend to reproduce the shapes of the **F**_es_-atoms in the directions of the covalent bonds, similar to the case from the preceding paragraph. According to Table 3 ▸, about 79.2–80.4% and 79.7–80.1% of the transferred ED are shared for N1 and N3, respectively.

The oxygen atoms of the hydroxyl groups O1 and O3, and of the carbonyl groups O2 and O4, act as electron occupiers with respect to their surroundings, since their ZFSs *S*(Ω) go further beyond the corresponding ZFSs *U*(Ω) and *P*(Ω) in all directions, thus expanding the electron-holding volumes and capturing the electrons held inside them (Figs. 8 ▸ and 9 ▸). The atoms are highly negatively charged. In the series from the ∇ρ-atom through the **F**_*k*_-pseudoatom to the **F**_es_-pseudoatom, the charges become closer to zero (Table 3 ▸), while their volumes gradually decrease, for example, from 15.67 through 11.54 to 6.18 Å^3^ (EMM), from 15.37 through 11.40 to 6.01 Å^3^ (TAAM) and from 15.38 through 11.14 to 5.71 Å^3^ (TMM) for O1; or from 16.83 through 12.83 to 6.04 Å^3^ (EMM), from 16.26 through 11.99 to 6.13 Å^3^ (TAAM) and from 16.56 through 12.57 to 5.92 Å^3^ (TMM) for O2. In contrast to N1 and N3, all the oxygen atoms are characterized by a noticeably smaller sharing of the captured ED: 63.9–67.5% for O1, 65.8–69.7% for O2, 63.8–66.8% for O3 and 64.7–66.9% for O4. This peculiarity is mainly due to the fact that a significant part of the entire captured ED is transferred as a result of the formation of noncovalent interactions (Kartashov *et al.*, 2023 ▸), in which the electrostatic nature is dominant or large. Indeed, Fig. 8 ▸ shows that, for the Lewis-type nonclassical hydrogen bonds [C5*^c^*—]H5*^c^* ⋯O2 and [C61*^c^*—]H61c*^c^* ⋯O2, only a small fraction of the transferred charge density, observed within the ρ-to-φ_es_-basin penetration gaps, are shared, that is, found within the ρ-to-φ_*k*_-basin gaps. For each of these interactions, the corresponding ZFS *P*(Ω) lies much closer to the ZFS *S*(Ω) than to the ZFS *U*(Ω), thus emphasizing the predominant electrostatic nature. For comparison, the polar bonds C6—N1 and C2—N3 or the very polar bonds C2=O2 and C4=O4 are all characterized by a tight pseudoatomic boundary-to-boundary adhesion, at least along the internuclear lines.

### 3.5. Applicability of transferable pseudo-atoms

We have recently performed an X-ray diffraction charge density study of another 6-methyl­uracil derivative, 1,6-di­methyl-3-(prop-2-yn-1-yl)pyrimidine-2,4(1*H*,3*H*)-dione (Shteingolts, Saifina *et al.*, 2021 ▸; Shteingolts, Stash *et al.*, 2021 ▸). We have constructed trajectory maps for this compound (Fig. S3) and compared them with those for 1,3-bis­(2-hy­droxy­ethyl)-6-methyl­pyrimidine-2,4(1*H*,3*H*)-dione studied here, and it is evident that not only the partially transferable atomic ρ-basins but also the pseudoatomic φ_es_- and φ_*k*_-basins are reproduced within a single structural fragment and similar environment, that is, the uracil heterocyclic moiety, in our case. This can be illustrated by comparing the maps in Figs. 8 ▸(*a*) and S3. This may mean that, by constructing a structural model from the appropriate multipole pseudo-atoms available in modern experimental and theory-based databases (Domagała *et al.*, 2012 ▸; Kumar *et al.*, 2019 ▸), it is possible to reconstruct the behavior of the force fields **F**_es_(**r**) and **F**_*k*_(**r**) and the associated pseudoatoms in crystals and even macromolecules, such as proteins and their receptor–ligand complexes. If so, then such easily and quickly obtained structural models can be used both for the study of complex polyatomic systems and for the analysis of crystals of small molecules, for which, for one reason or another, diffraction data of sufficiently high resolution and quality are not available.

To validate the applicability of transferable aspherical pseudo-atoms for restoring the electronic force density fields **F**_es_(**r**), **F**_*k*_(**r**) and 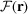 , we have prepared the structural model (TAAM) with parameters adopted from UBDB2018 (Volkov *et al.*, 2004 ▸; Kumar *et al.*, 2019 ▸) for the uracil derivative crystal and compared it with EMM and TMM. Figs. 8 ▸ and 9 ▸ help visually collate the force behavior within various covalent and noncovalent bonding regions. The pseudoatomic charges are compared in Table 3 ▸. To our satisfaction, the charge, shape and volume of the pseudoatoms and the relative arrangement of the ZFSs *S*(Ω), *U*(Ω) and *P*(Ω) obtained using the TAAM model show a great similarity to those obtained by EMM and TMM.

**Figure 9:**
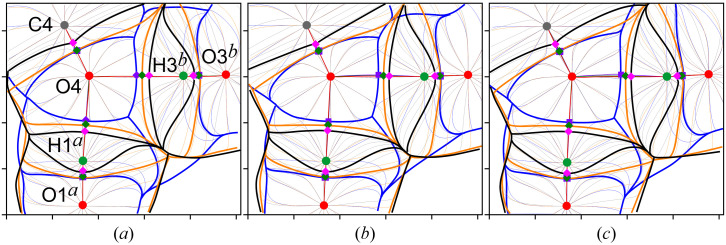
Superpositions of the trajectories of the (*a*) experimental, (*b*) theoretical or (*c*) semi-theoretical inner-crystal gradient vector fields of the ED ∇ρ(**r**) (black), the electrostatic potential ∇φ_es_(**r**) (blue) and the kinetic potential ∇φ_*k*_(**r**) (orange) of 1,3-bis­(2-hy­droxy­ethyl)-6-methyl­uracil in the plane of two classical hydrogen bonds. The compared maps are calculated based on (*a*) the experimental pseudo-atom model (EMM) or the aspherical pseudo-atom models with parameters (*b*) fitted to the theoretical structure factors (TMM) or (*c*) taken from the database (TAAM). (Pseudo)atomic boundaries are highlighted with thicker lines of the corresponding color. Saddle CPs (3, −1) in ρ(**r**), φ_es_(**r**) and φ_*k*_(**r**) are marked by magenta and violet rhombuses and green squares, respectively. Gradient paths starting from these points and ending at the nuclei are colored red, violet and green, respectively. An out-of-plane distance is set to 0.3 Å. The distance between axis tick marks is 1 Å. The trajectory maps are plotted on the same atomic plane of O4, H3*^b^* and H1*^a^*.

Consider, for example, the carbonyl O4 oxygen atom involved in the two classical hydrogen bonds (Fig. 9 ▸). Recall that formally these hydrogen bonds are not formed by the pure Lewis mechanism, since O4 does not deliver its LPs to the binding internuclear regions [Figs. 7 ▸(*c*) and 7 ▸(*d*)]. However, the models reveal that its ρ-basin invades the adjacent pseudoatomic φ_es_- and φ_*k*_-basins, thus occupying a portion of the ED held within the ρ-to-φ_es_-basin overlapping gaps, including that associated with H1*^a^* and H3*^b^*. The ∇ρ-atom O4 is highly negatively charged (Table 3 ▸) and acts as an occupier. The **F**_*k*_-pseudoatom O4 is also quite negatively charged; it has the intermediate volume of 12.34 Å^3^ for EMM, 12.68 Å^3^ for TAAM and 12.30 Å^3^ for TMM between the ∇ρ-atom O4 (17.14 Å^3^ for EMM, 16.82 Å^3^ for TAAM and 16.80 Å^3^ for TMM) and the **F**_es_-pseudoatom O4 (5.57 Å^3^ for EMM, 6.28 Å^3^ for TAAM and 5.78 Å^3^ for TMM). Regardless of the model, for each classical hydrogen bond, the ZFS *P*(Ω) takes an intermediate position between *U*(Ω), which is closer to the nucleus O4, and *S*(Ω), which is closer to the nucleus H1*^a^* or H3*^b^* along the bond and binding paths.

Finally, we note a difference between the models that is important in the context of the quantum topological binding approach. While the bond ρ- and binding φ_es_- and φ_*k*_-paths of the covalent and classical hydrogen bonds for all models essentially coincide with the corresponding internuclear straight lines (Figs. 8 ▸ and 9 ▸), the trajectories of the curved ρ- and φ-paths of the nonclassical hydrogen bonds do not match. Moreover, the φ_es_-path disappears in the case of [C61*^c^*—]H61c*^c^* ⋯O2 [Fig. 8 ▸(*b*)]. Therefore, the bond ρ- and binding φ_es_- and φ_*k*_-paths should be analyzed with caution for weak noncovalent interactions.

### 3.6. Binding and bonding within the free hydrogen-bonded and π-stacked dimer

**Figure 10:**
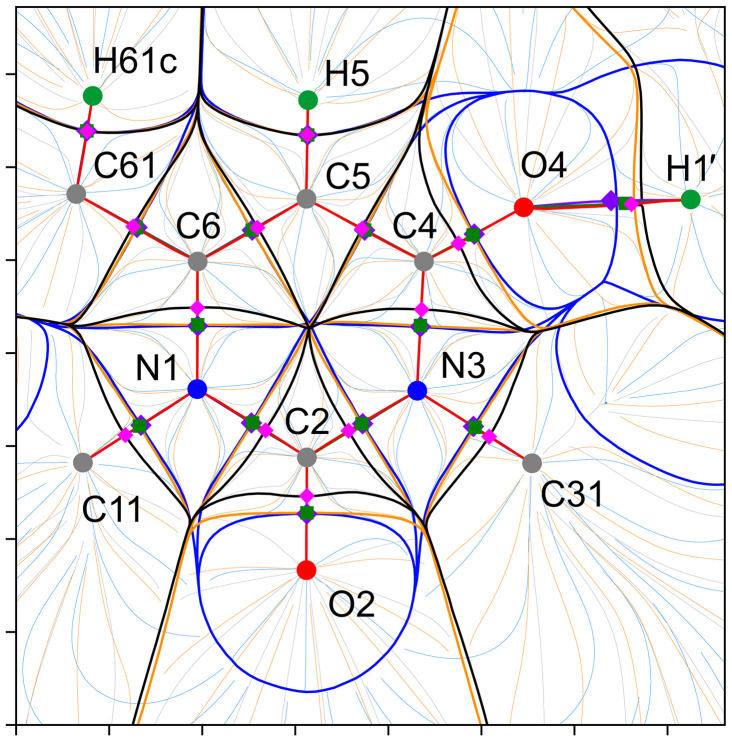
Overlay of theoretical trajectory maps of the gradients of the ED ∇ρ(**r**) (black), the electrostatic potential ∇φ_es_(**r**) (blue) and the kinetic potential ∇φ_*k*_(**r**) (orange) of the optimized hydrogen-bonded, π-stacked dimer of 1,3-bis­(2-hy­droxy­ethyl)-6-methyl­pyrimidine-2,4(1*H*,3*H*)-dione, representing the uracil fragment. Zero-flux surfaces are highlighted with thicker lines of the corresponding color. The fields are shown as trajectories and superimposed in the planes of N1, N3 and C6. Saddle CPs (3, −1) in ρ(**r**), φ_es_(**r**) and φ_*k*_(**r**) are indicated by magenta and violet rhombuses and green squares. Gradient lines emerging from these CPs and ending at the nuclei are colored red, violet and green, respectively. An out-of-plane distance is set to 1 a.u. The distance between axis tick marks is 1 Å.

To further investigate the features of chemical bonding in supramolecular systems using the quantum potentials and in terms of the quantum-topological binding approach, the structure of the dimer shown in Fig. 3 ▸(*a*) was extracted from the crystal and then theoretically optimized. The optimized dimer geometry is visualized in Fig. S4. The wavefunction-derived data for the dimer (Fig. 10 ▸) suggest a great similarity in the force-field features within the heterocyclic uracil moiety compared with the multipole data for the crystal (Figs. 8 ▸ and S2).

**Figure 11:**
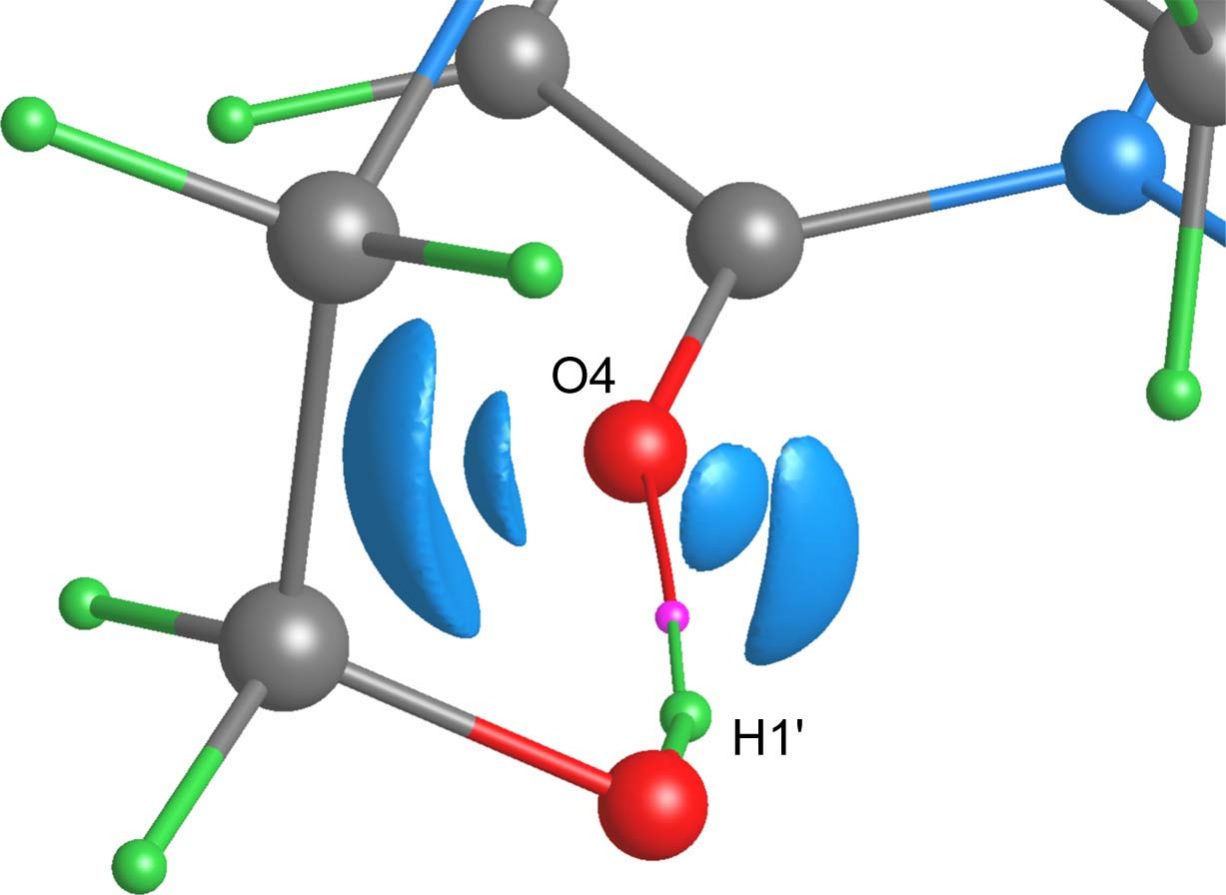
Isosurfaces of the fermionic potential φ_f_(**r**) at −0.05 a.u. of the free hydrogen-bonded, π-stacked dimer of 1,3-bis­(2-hy­droxy­ethyl)-6-methyl­uracil, calculated around the O4 atom using the wavefunction. The BCP for the hydrogen bond H1′⋯O4 is shown as a magenta-colored sphere and the associated bond path is shown as a thin line.

Although the cyclic hydrogen-bond motif is retained, an important change in the geometry of the hydrogen bond is observed upon optimization: *d*_H⋯A_ = 1.80916 Å, *d*_D⋯A_ = 2.74473 Å, 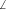 DHA = 161.583°, α_Hb_ = 25.52°, β_Hb_ = 9.77° for H1′⋯O4 (compare with the data in Table 1 ▸). The calculated values of ρ_b_ and ∇^2^ρ_b_ are 0.220 e Å^–3^ and 2.520 e Å^–5^; the approximate interaction energies 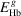 and 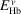 are equal to 7.682 and 9.704 kcal mol^–1^, respectively. All this indicates favorable geometric parameters and the approximate directionality of the hydrogen bond H1′⋯O4 toward one of the two LPs of the carbonyl O4 oxygen atom in the gas-phase dimer. The location of the LPs of O4, associated with the regions of negative φ_f_(**r**), in the plane of the heterocyclic fragment is evident from Fig. 11 ▸. The important detail, which we have already discussed for other compounds (Kartashov *et al.*, 2023 ▸; Saifina *et al.*, 2023 ▸), follows from the figure, namely that the relative volume enclosed by the isosurface of φ_f_(**r**), corresponding to the electron pair involved in the interaction H1′⋯O4, decreases significantly. This behavior of φ_f_(**r**) is promising for the description of the Lewis-type interaction mechanism.

**Figure 12:**
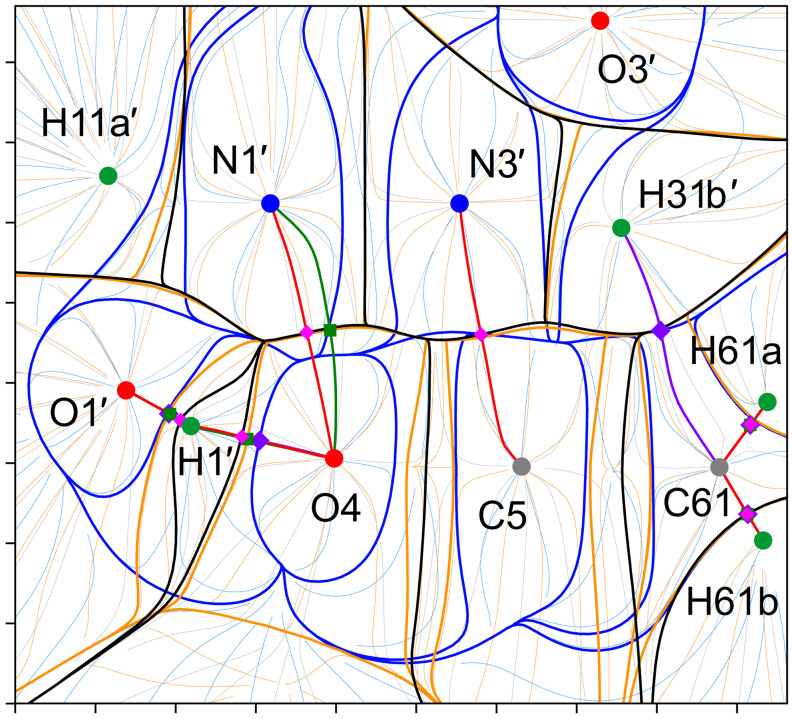
Theoretical vector fields of the gradients of the ED ∇ρ(**r**) (black), the electrostatic potential ∇φ_es_(**r**) (blue) and the kinetic potential ∇φ_*k*_(**r**) (orange) of the free hydrogen-bonded, π-stacked dimer of 1,3-bis­(2-hy­droxy­ethyl)-6-methyl­pyrimidine-2,4(1*H*,3*H*)-dione, representing the uracil–uracil interaction. Zero-flux surfaces are highlighted with thicker lines of the corresponding color. The fields are shown as trajectories and overlaid in the planes of the N1′, N3′ and O4 atoms. Saddle CPs (3, −1) in ρ(**r**), φ_es_(**r**) and φ_*k*_(**r**) are indicated by magenta and violet rhombuses and green squares, respectively. Gradient lines starting from these CPs and ending at the nuclei are colored red, violet and green, respectively. An out-of-plane distance is set to 0.5 a.u. The distance between the axis tick marks is 1 Å.

Although the mechanism of the hydrogen bond formation in the dimer is changed and can be described as a donor–acceptor one with the O4 oxygen atom as an LP donor, as can be seen from Figs. 10 ▸ and 12 ▸, the order of the ZFSs crossing the H1′⋯O4 bond path remains the same, with *U*(Ω) and *P*(Ω) inside the ρ-basin of O4, that is, the roles of the hydrogen and oxygen atoms are preserved as a contributor and an occupier, respectively (Fig. 9 ▸). Moreover, the roles of hydrogen and oxygen atoms is the same as in the hydrogen bonds in the previously studied compounds (Shteingolts *et al.*, 2022 ▸; Kartashov *et al.*, 2023 ▸). The interaction H1′⋯O4 is characterized by the complete set of bond ρ- and binding φ_es_- and φ_*k*_-paths. However, we note that the ZFS *P*(Ω) is located closer to the ZFS *S*(Ω) than *U*(Ω) along the H⋯O bond path (Fig. 12 ▸), which, in turn, may indirectly indicate a more electrostatic nature of the bond compared with that in the crystal (Fig. 9 ▸); at the same time, such a comparison is limited due to physical differences between multipole and wavefunction derived models. Anyway, the ZFS arrangement along H1′⋯O4, with *P*(Ω) showing a distinct lag from *S*(Ω) within the entire interatomic region (Fig. 12 ▸), indicates its more covalent character compared with the other intermolecular interactions within the dimer. Notably, the ∇ρ-atom and **F**_*k*_-pseudoatom of O4 in a free molecule are characterized by open ρ- and φ_*k*_-basins, while the nucleus of the respective **F**_es_-pseudoatom is screened by the hemispherical closed φ_es_-basin similar to that of O2 in the dimer (Fig. 10 ▸). The appearance of the attractor H1′ resulting from the dimer formation leads to both the closure of the pseudoatomic φ_*k*_-basin of O4 and to the flattening of the **F**_es_-atom from the side of the hydrogen bond H1′⋯O4 (Figs. 10 ▸ and 12 ▸). Comparing O1–H1 and H1′⋯O4 or N1′⋯O4 allows us to demonstrate the differences between a polar covalent bond with *P*(Ω) adjacent to *U*(Ω) at least along the bond path and a noncovalent interaction with *P*(Ω) somewhere between *U*(Ω) and *S*(Ω) or even adjacent to *S*(Ω) (Fig. 12 ▸).

**Figure 13:**
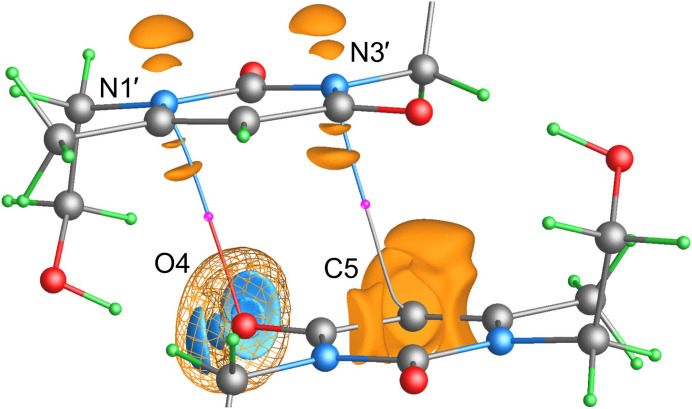
Isosurfaces of the fermionic potential φ_f_(**r**) at −0.05 a.u. (blue) and 0.1 a.u. (orange) of the free hydrogen-bonded, π-stacked dimer of 1,3-bis­(2-hy­droxy­ethyl)-6-methyl­uracil, calculated around the labeled atoms using the wavefunction. BCPs for the interactions N3′⋯C5 and N1′⋯O4 are shown as magenta spheres and the associated bond paths are shown as thin lines.

Another important geometric change is observed in the mutual arrangement of the heterocyclic fragments: the centroid-to-centroid distance decreases slightly from 3.8844 (1) to 3.87992 Å, while the interplanar distance decreases from 3.3660 (1) to 3.25641 Å. According to the QTAIM analysis, these lead to the appearance of two almost symmetrical pairs of heteroatomic intermolecular interactions of N⋯C and N⋯O types which, in turn, form the π⋯π interaction (Figs. 13 ▸ and S4). The Lewis mechanism of their formation turned out to be remarkable. The φ_f_(**r**) isosurfaces associated with these interactions are shown in Fig. 13 ▸. The carbonyl O4 oxygen atom exhibits a torus of reduced φ_f_(**r**) at an isovalue of 0.1 a.u., within which the deeply negative lobes associated with two LPs can be identified. The LPs are located in the plane of the amide fragment. As mentioned above, one of the LPs is involved in the hydrogen bonding with the H1′ atom. Each nitro­gen atom features regions of decreased φ_f_(**r**) above and below the heterocycle at the same isovalue of 0.1 a.u., which could be related to the π-density. Note that the LPs of the oxygen atom, as well as the π-density of the nitro­gen atoms on either side of the plane, are expressed as doublets of low-potential localization domains; the fermionic potential φ_f_(**r**) obtained from the multipole models lacks such a detail (Figs. 6 ▸ and 7 ▸). Along the bond path N3′⋯C5, the lump of decreased φ_f_(**r**) (π-density) at the Lewis base N3′ is directed toward the region of enhanced φ_f_(**r**) (vacancy) at the Lewis acid C5, thus forming the tetrel N3′⋯C5 interaction. Similarly, along the bond path N1′⋯O4, there is a lump of decreased φ_f_(**r**) at N1′ directed toward the aforementioned torus at O4—specifically, toward the bifurcated site, within which the potential is locally enhanced compared with the two reduced lobes. Interestingly, the comparable mechanism of the N⋯O[=C] interaction was previously studied by us in the crystal of 1,6-di­methyl-3-(prop-2-yn-1-yl)pyrimidine-2,4(1*H*,3*H*)-dione (Shteingolts, Saifina *et al.*, 2021 ▸; Shteingolts, Stash *et al.*, 2021 ▸). We assume that the atoms N1′ and O4 behave as a Lewis base and a Lewis acid, respectively, for the interaction N1′⋯O4. Nevertheless, the role of O4 as a Lewis acid is still controversial.

Both interactions N3′⋯C5 and N1′⋯O4 with *P*(Ω) adjacent to *S*(Ω) are primarily electrostatic in nature. The N1′⋯O4 interactions are characterized by the absence of the φ_*k*_-path, whereas no binding paths are observed for N3′⋯C5 (Fig. 12 ▸). Recall that the criterion for categorizing noncovalent interactions proposed by Bartashevich *et al.* (2019 ▸), under reformulation by Saifina *et al.* (2023 ▸), states that an electron occupier usually carries a nucleophilic site and acts as a Lewis base, whereas an electron contributor often provides its electrophilic site and acts as a Lewis acid. As can be seen from Figs. 12 ▸ and S5, the pseudoatomic φ_es_-basin of N3′ or N1′ extends forward, permeating into the ρ-basin of C5 or O4, thus contributing a portion of the ED contained in the ρ-to-φ_es_-basin penetration gap. Therefore, the nitro­gen atoms in the interactions N3′⋯C5 and N1′⋯O4 are electron contributors, or in other words, electropositive along the corresponding directions (Saifina *et al.*, 2023 ▸). At the same time, they are more likely to act as Lewis bases, which contradicts the above criterion.

To this end, we can conclude that it is rather unsound to identify nucleophilic and electrophilic sites from the relative arrangement of the atomic and pseudoatomic ZFSs *S*(Ω), *U*(Ω) and *P*(Ω) without the direct location of the LP (or analog) involved in an interaction. In connection with the above, we believe it is necessary to extend the widely used classification of noncovalent interactions, in which the element type of a Lewis acid is noted, by designating the role of this atom in the interatomic charge transfer. For instance, H1′⋯O4 is an electron-contributor-hydrogen bond or N3′⋯C5 is an electron-occupier-tetrel bond.

## 4. Conclusions

The inner-crystal and inner-supramolecular electronic, potential and force-field structural levels of 1,3-bis­(2-hy­droxy­ethyl)-6-methyl­pyrimidine-2,4(1*H*,3*H*)-dione were studied using high-resolution single-crystal X-ray diffraction and computational theoretical methods. Among other intermolecular interactions involved in the crystal formation, two classical and two nonclassical hydrogen bonds, [O—]H⋯O[=C] and [C—]H⋯O[=C], were distinguished by considering the static potential acting on an electron in a molecule φ_em_(**r**) and the magnitude of the associated total static force 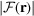 , both heat-mapped on the atomic surfaces. They were further considered within the concepts of inter­atomic charge transfer and electron LP donation–acceptance. So, the interatomic charge transfer and the subsequent sympathetic quantum-chemical response, which is associated with the sharing of the transferred ED, were investigated within the quantum-topological binding approach based on the force density fields—specifically, by means of considering the penetration of the ∇ρ-atom of the occupier into the neighboring electrostatic and kinetic **F**_es_- and **F**_*k*_-force field pseudoatoms of the contributor. The von Weizsäcker (bosonic) potential φ_W_(**r**) and the fermionic potential φ_f_(**r**) were used as functions for the LP and π-density location. Although the two nonclassical hydrogen bonds considered are formed by the expected donor–acceptor or Lewis-type mechanism, the two classical hydrogen bonds in the uracil derivative crystals are formed by a formally different mechanism because these interactions are not directed to one of the LPs of the carbonyl oxygen atom. Nevertheless, the oxygen and hydrogen atoms in all four hydrogen bonds act as electron occupiers and contributors, respectively.

The geometry optimization of the hydrogen-bonded cyclic dimer isolated from the uracil derivative crystal led to the return of the conventional Lewis-type formation mechanism for the classical hydrogen bond, with the carbonyl oxygen atom acting simultaneously as a Lewis base and as an electron occupier. Furthermore, the optimization resulted in the appearance of two nearly symmetrical pairs of heteroatomic intermolecular interactions of the N⋯O and N⋯C types, which, in turn, constitute the π⋯π interaction between the uracil moieties. Remarkably, the nitro­gen atoms in these interactions behave rather like a Lewis base and an electron contributor, that is, otherwise than in the hydrogen bond mentioned above. Thus, it has been argued that the identification of a Lewis base and a Lewis acid within a noncovalent interaction from the relative positions of the atomic and pseudoatomic zero-flux surfaces along an internuclear region is rather unreliable.

We believe that describing chemical interactions in terms of the donor–acceptor Lewis mechanism by identifying the nucleophilic site and its corresponding vacancy, as well as the description of the ρ-to-φ_es_-basin penetration gaps in terms of atom partial positioning of an atom in the electrophilic influence zone of a neighboring atom, is useful but largely arbitrary and non-universal. The binding approach based on force density fields lacks such drawbacks and is applicable to covalent and noncovalent interactions, as well as to nonbonded contacts. This approach makes it possible to follow the interatomic charge redistribution determined by the classical electrostatic effect and the response of the system to the electron transfer, induced by both quantum static and kinetic effects. We also suggest that when describing polar interatomic interactions within orbital-free considerations, it makes more physical sense to identify electronegative (electron occupier) and electropositive (electron contributor) atoms or subatomic fragments rather than nucleophilic and electrophilic sites.

Finally, it was found that not only the quantum-topological atoms but also the force-field pseudoatoms are reproduced within a single structural fragment and a similar environment and can thus be considered partially spatially transferable. Using the uracil derivative crystal as an example, transferable multipole pseudo-atoms adopted from the database were found to be applicable to the reconstruction of the inner-crystal electronic force density fields. The model built from the transferable pseudo-atoms was shown to reproduce the general behavior of the vector fields ∇ρ(**r**), **F**_es_(**r**) and **F**_*k*_(**r**); the expected distinctive features of the quantum scalar fields of φ_W_(**r**), φ_f_(**r**) and φ_em_(**r**); and the characteristics of the force-field pseudoatoms, such as charge, shape and volume; as well as to replicate the relative arrangement of the atomic *S*(Ω) and pseudoatomic *U*(Ω) and *P*(Ω) zero-flux surfaces along covalent bonds and noncovalent interactions. However, it was found that the trajectories of the curved bond ρ- and binding φ-paths of some noncovalent interactions may not match in the different models. Thus, such models with transferable pseudo-atoms could be applied to study chemical bonding and binding in complex many-electron multinuclear systems.

## Supplementary Material

Crystal structure: contains datablock(s) I. DOI: 10.1107/S2052252523007108/lt5061sup1.cif

Structure factors: contains datablock(s) saif004_m. DOI: 10.1107/S2052252523007108/lt5061sup2.hkl

Supporting figures and tables. DOI: 10.1107/S2052252523007108/lt5061sup3.pdf

CCDC reference: 2259862

## Figures and Tables

**Table 1:** Geometric parameters of the hydrogen bonds studied in the crystal of 1,3-bis­(2-hy­droxy­ethyl)-6-methyl­uracil The units of measurement are as follows: interatomic distances *d*_H⋯A_ and *d*_D⋯A_ (Å); angles 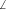 DHA, α_Hb_ and β_Hb_ (°). The first and second rows for each bond contain the data obtained from the experimental multipole model (EMM) or the aspherical pseudo-atom model with parameters taken from the database (TAAM), whereas the data for the third row are taken from the optimized crystal structure (TM).

D—H⋯A		*d*_H⋯A_	*d*_D⋯A_	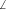 DHA	α_Hb_	β_Hb_
O1*^a^*—H1*^a^* ⋯O4	EMM	1.8537 (29)	2.81945(15)	170.41 (9)	6.21	30.90
	TAAM	1.8708 (25)	2.81915(15)	170.12 (8)	6.51	30.46
	TM	1.83711	2.80882	172.915	4.27	28.91
O3*^b^*—H3*^b^* ⋯O4	EMM	2.0504 (30)	2.99035 (17)	162.43 (16)	18.56	62.77
	TAAM	2.0665 (27)	2.98988 (16)	161.31 (15)	17.80	62.35
	TM	2.03917	2.99836	167.364	23.35	62.92
C5*^c^*—H5*^c^* ⋯O2	EMM	2.23628	3.25747 (16)	156.504	22.07	0.38
	TAAM	2.23664	3.25783 (16)	156.504	22.07	0.38
	TM	2.22835	3.25791	158.170	21.39	1.17
C61*^c^*—H61c*^c^* ⋯O2	EMM	2.52172	3.53521 (18)	154.056	35.91	0.98
	TAAM	2.52218	3.53573 (18)	154.067	35.91	0.98
	TM	2.51980	3.53239	153.902	36.07	0.82

**Table 2:** Selected characteristics of the BCPs of the hydrogen interactions in the crystal of 1,3-bis­(2-hy­droxy­ethyl)-6-methyl­uracil The units of measurement are as follows: nucleus-to-BCP distances *d*_H⋯b_ and *d*_A⋯b_ (Å); ED ρ_b_ (e Å^−3^) and its Laplacian 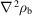 (e Å^−5^); kinetic and potential electronic energy densities *g*_b_ and *v*_b_ (a.u.); and estimated interaction energies 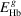 and 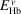 (kcal mol^−1^). Subscript b indicates that the property is calculated at the BCP. Topological data are obtained for ED calculated from the wavefunction (TM) or reconstructed from the experimental pseudo-atom model (EMM) or the aspherical pseudo-atom models with parameters taken from the database (TAAM) or fitted to the theoretical structure factors (TMM).

D—H⋯A		*d*_H⋯b_	*d*_A⋯b_	ρ_b_	∇^2^ρ_b_	*g*_b_ × 10^2^	*v*_b_ × 10^2^	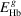	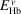
O1*^a^*—H1*^a^* ⋯O4	EMM	0.643	1.211	0.190	1.636	1.881	−2.065	5.064	6.480
	TAAM	0.676	1.195	0.199	1.817	2.064	−2.243	5.556	7.037
	TMM	0.663	1.174	0.214	2.067	2.338	−2.532	6.293	7.945
	TM	0.669	1.190	0.189	2.757	2.766	−2.672	7.446	8.384
O3*^b^*—H3*^b^* ⋯O4	EMM	0.749	1.303	0.124	1.089	1.123	−1.116	3.024	3.502
	TAAM	0.783	1.285	0.131	1.236	1.256	−1.230	3.381	3.858
	TMM	0.764	1.275	0.134	1.444	1.414	−1.330	3.805	4.173
	TM	0.756	1.306	0.122	1.848	1.659	−1.401	4.467	4.396
C5*^c^*—H5*^c^* ⋯O2	EMM	0.887	1.362	0.072	1.361	1.090	−0.767	2.934	2.405
	TAAM	0.902	1.335	0.097	1.153	1.041	−0.886	2.803	2.779
	TMM	0.887	1.351	0.073	1.380	1.106	−0.780	2.977	2.447
	TM	0.884	1.362	0.085	1.345	1.088	−0.780	2.928	2.447
C61*^c^*—H61c*^c^* ⋯O2	EMM	1.044	1.512	0.036	0.672	0.513	−0.328	1.380	1.029
	TAAM	1.060	1.465	0.054	0.628	0.526	−0.400	1.415	1.254
	TMM	1.051	1.488	0.040	0.709	0.546	−0.356	1.469	1.117
	TM	1.029	1.510	0.052	0.683	0.551	−0.394	1.485	1.237

**Table 3:** Atomic and pseudoatomic charges in crystalline 1,3-bis­(2-hy­droxy­ethyl)-6-methyl­uracil The atomic and pseudoatomic charges *q* are given in e. The subscripts ∇ρ and **F**_*k*_ indicate that the property is integrated over the ∇ρ-atom and **F**_*k*_-pseudoatom, respectively. The integration data are obtained for ED reconstructed from the experimental pseudo-atom model (EMM) or the aspherical pseudo-atom models with parameters taken from the database (TAAM) or fitted to the theoretical structure factors (TMM).

(Pseudo)atom	EMM		TAAM		TMM	
	*q*_∇ρ_	*q*_**F**_*k*__	*q*_∇ρ_	*q*_**F**_*k*__	*q*_∇ρ_	*q*_**F**_*k*__
O1	−0.967	−0.314	−0.909	−0.315	−0.919	−0.332
O2	−1.136	−0.345	−1.110	−0.349	−1.000	−0.342
O3	−0.939	−0.312	−0.898	−0.314	−0.895	−0.324
O4	−1.187	−0.392	−1.056	−0.333	−1.020	−0.360
N1	−0.988	−0.193	−0.966	−0.168	−0.843	−0.175
N3	−1.026	−0.204	−0.987	−0.172	−0.838	−0.170
C2	1.585	0.253	1.616	0.284	1.402	0.289
C4	1.192	0.230	1.210	0.209	1.039	0.232
C5	−0.070	−0.081	−0.078	−0.038	−0.037	−0.063
C6	0.300	0.096	0.338	0.086	0.253	0.103
C11	0.124	0.094	0.324	0.079	0.212	0.101
C12	0.365	0.126	0.412	0.114	0.369	0.119
C31	0.192	0.092	0.327	0.081	0.199	0.083
C32	0.328	0.113	0.416	0.119	0.363	0.124
C61	−0.096	−0.018	0.136	−0.004	0.037	0.003
H1	0.629	0.225	0.536	0.188	0.534	0.176
H3	0.600	0.225	0.528	0.190	0.518	0.179
H5	0.103	0.030	0.056	0.052	0.083	0.038
H11a	0.113	0.042	0.013	0.024	0.065	0.039
H11b	0.151	0.059	0.010	0.024	0.071	0.034
H12a	0.038	0.006	0.011	0.023	0.050	0.032
H12b	0.090	0.031	0.020	0.030	0.036	0.023
H31a	0.130	0.060	0.011	0.023	0.080	0.043
H31b	0.070	0.034	0.020	0.030	0.077	0.044
H32a	0.094	0.034	0.015	0.026	0.023	0.015
H32b	0.045	0.004	0.010	0.025	0.040	0.024
H61a	0.072	0.023	−0.001	0.029	0.053	0.033
H61b	0.109	0.048	−0.002	0.025	0.029	0.017
H61c	0.091	0.042	0.010	0.039	0.032	0.026
