# Supplementary material for: Applicability of transferable multipole pseudo-atoms for restoring inner-crystal electronic force density fields. Chemical bonding and binding features in the crystal and dimer of 1,3-bis­(2-hy­droxy­ethyl)-6-methyl­uracil
Source: IUCrJ. 2023 Sep 1;10(Pt 5):584–602. doi: 10.1107/S2052252523007108 (PMC10478519; doi:10.1107/S2052252523007108)
Supplement: Supplementary file 3 [file m-10-00584-sup3.pdf]

IUCrJ

Volume 10 (2023)

Supporting information for article:

Applicability of transferable multipole pseudo-atoms for restoring inner-crystal electronic force density fields. Chemical bonding and binding features in the crystal and dimer of 1,3-bis(2-hydroxyethyl)-6-methyluracil This article is part of IUCr2023.

Alina F. Saifina, Sergey V. Kartashov, Liliya F. Saifina and Robert R. Fayzullin

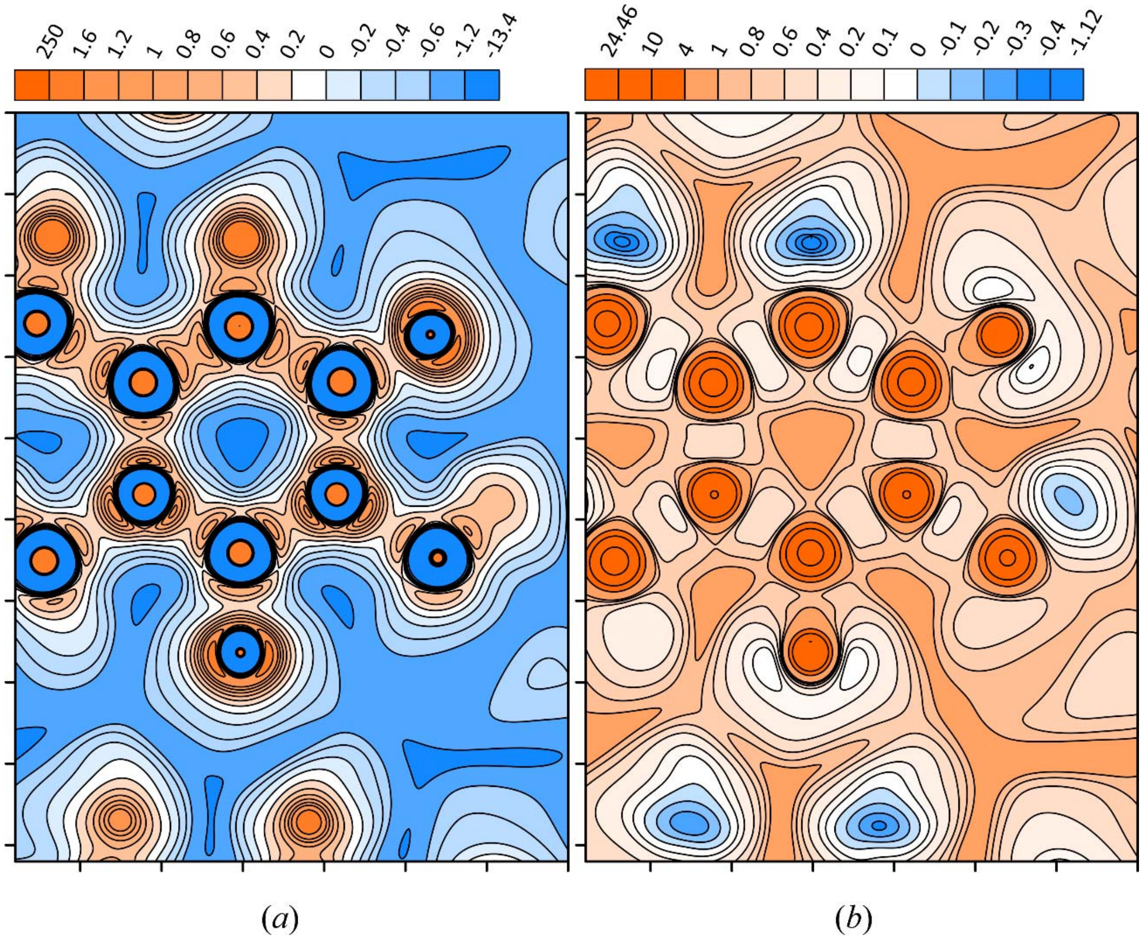

Figure S1 Contour maps of the inner-crystal (*a*) bosonic and (*b*) fermionic potentials, $\varphi_{\mathrm{W}}(\mathbf{r})$ and $\varphi_{\mathrm{f}}(\mathbf{r})$, for 1,3-bis(2-hydroxyethyl)-6-methyluracil, calculated from the transferable aspherical pseudo-atom model (TAAM). Contour intervals of 0.2 a.u. are used; additional contours are shown at –1.2 and 1.6 a.u. for $\varphi_{\mathrm{W}}(\mathbf{r})$ and –0.3, ±0.1, 4, 10 a.u. for $\varphi_{\mathrm{f}}(\mathbf{r})$. Function scale bars are displayed above the maps. The distance between adjacent axis tick marks is 1 Å. Both maps are plotted on the same atomic plane of N1, N3, and C6.

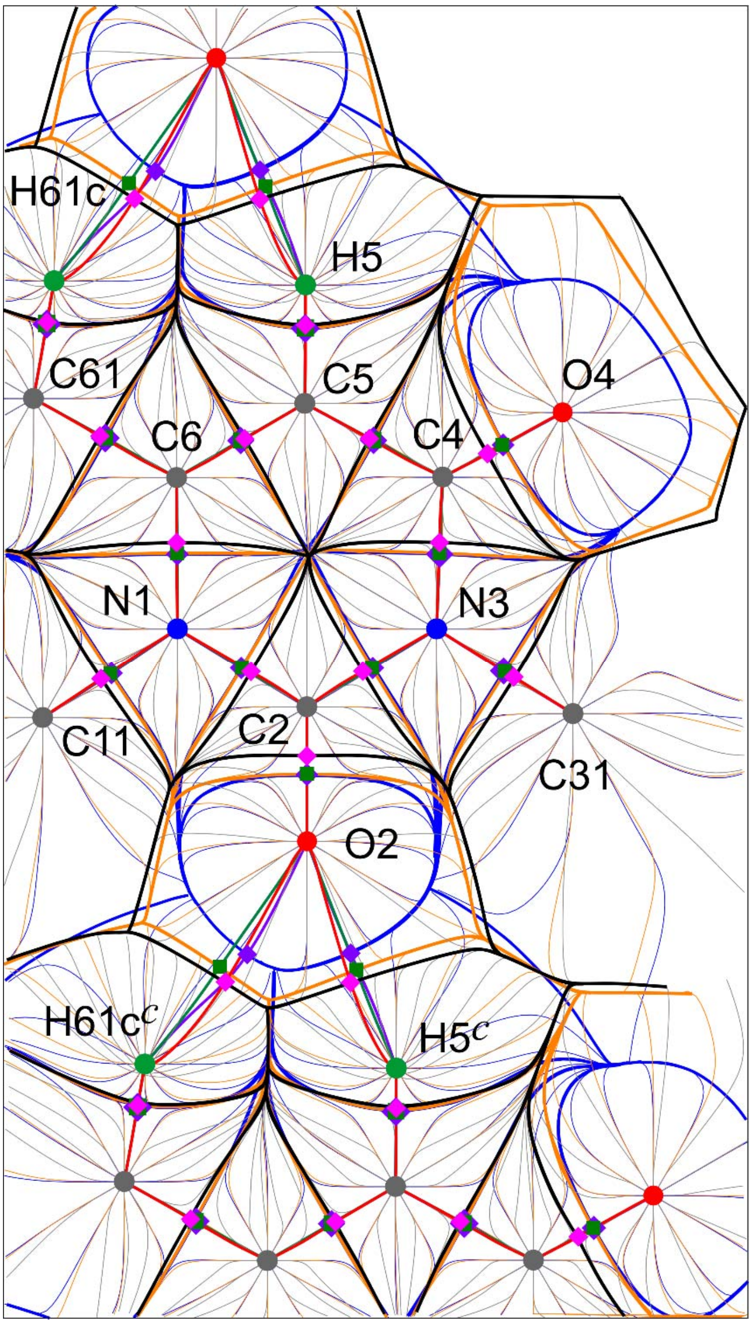

Figure S2 Superposition of the theoretical inner-crystal gradient fields of the electron density $\nabla\rho(\mathbf{r})$ (black), the electrostatic potential $\nabla\varphi_{es}(\mathbf{r})$ (blue), and the kinetic potential $\nabla\varphi_{k}(\mathbf{r})$ (orange) for the uracil core of 1,3-bis(2-hydroxyethyl)-6-methylpyrimidine-2,4(1*H*,3*H*)-dione. The maps are calculated based on the aspherical pseudo-atom model with the parameters fitted to the theoretical structure factors (TMM). (Pseudo)atomic boundaries are highlighted with thicker lines of the corresponding color. Saddle critical points (3, –1) in $\rho(\mathbf{r})$, $\varphi_{es}(\mathbf{r})$, and $\varphi_{k}(\mathbf{r})$ are indicated by magenta and violet rhombuses and green squares, respectively; the maximum critical points (3, –3) are shown as element-type-colored circles. Gradient paths connecting these critical points are colored red, violet, and green, respectively. An out-of-plane distance is set to 0.3 Å. The trajectory maps are plotted on the same atomic plane of N1, N3, and C6.

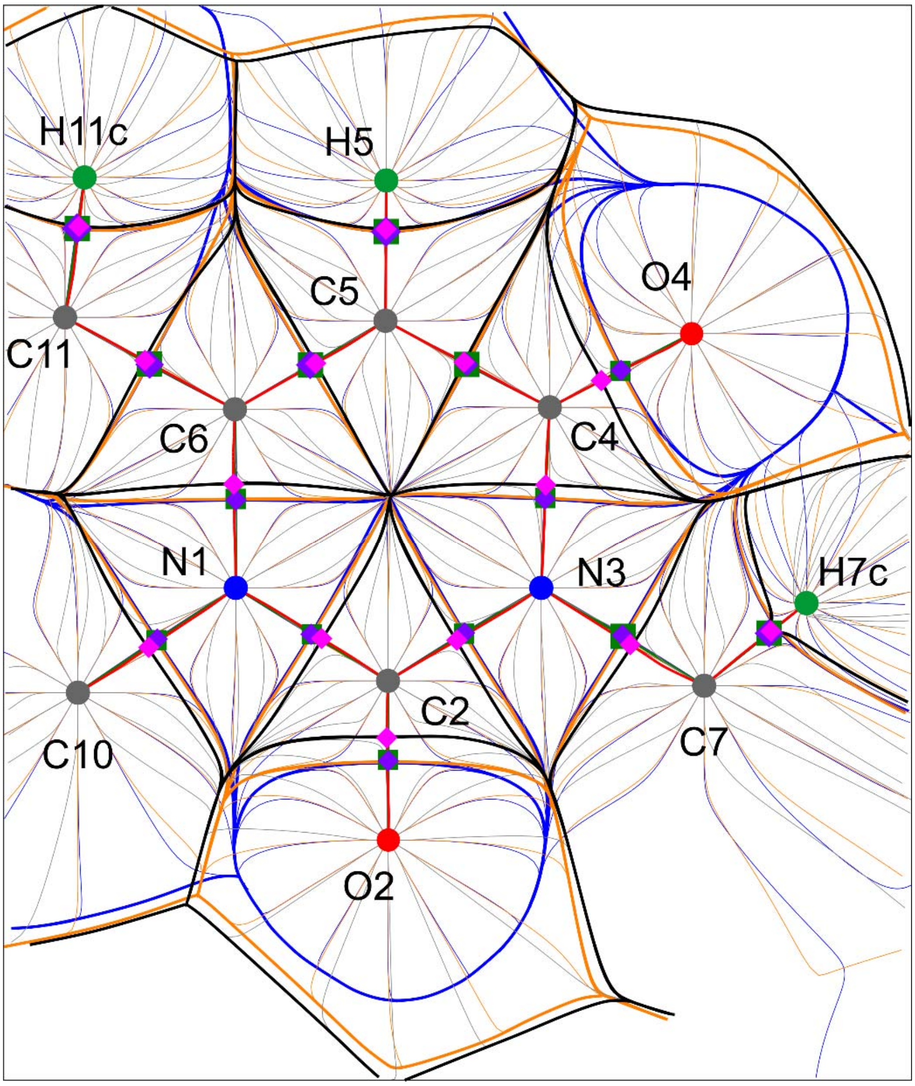

Figure S3 Superposition of the experimental inner-crystal gradient fields of the electron density $\nabla\rho(\mathbf{r})$ (black), the electrostatic potential $\nabla\varphi_{\mathrm{es}}(\mathbf{r})$ (blue), and the kinetic potential $\nabla\varphi_k(\mathbf{r})$ (orange) for the uracil core of 1,6-dimethyl-3-(prop-2-yn-1-yl)pyrimidine-2,4(1*H*,3*H*)-dione studied previously (Shteingolts, Saifina *et al.*, 2021; Shteingolts, Stash *et al.*, 2021). (Pseudo)atomic boundaries are highlighted with thicker lines of the corresponding color. Saddle critical points (3, –1) in $\rho(\mathbf{r})$, $\varphi_{\mathrm{es}}(\mathbf{r})$, and $\varphi_k(\mathbf{r})$ are indicated by magenta and violet rhombuses and green squares, respectively; the maximum critical points (3, –3) are shown as element-type-colored circles. Gradient paths connecting these critical points are colored red, violet, and green, respectively. An out-of-plane distance is set to 0.3 Å. The trajectory maps are plotted on the atomic plane of N1, N3, and C6.

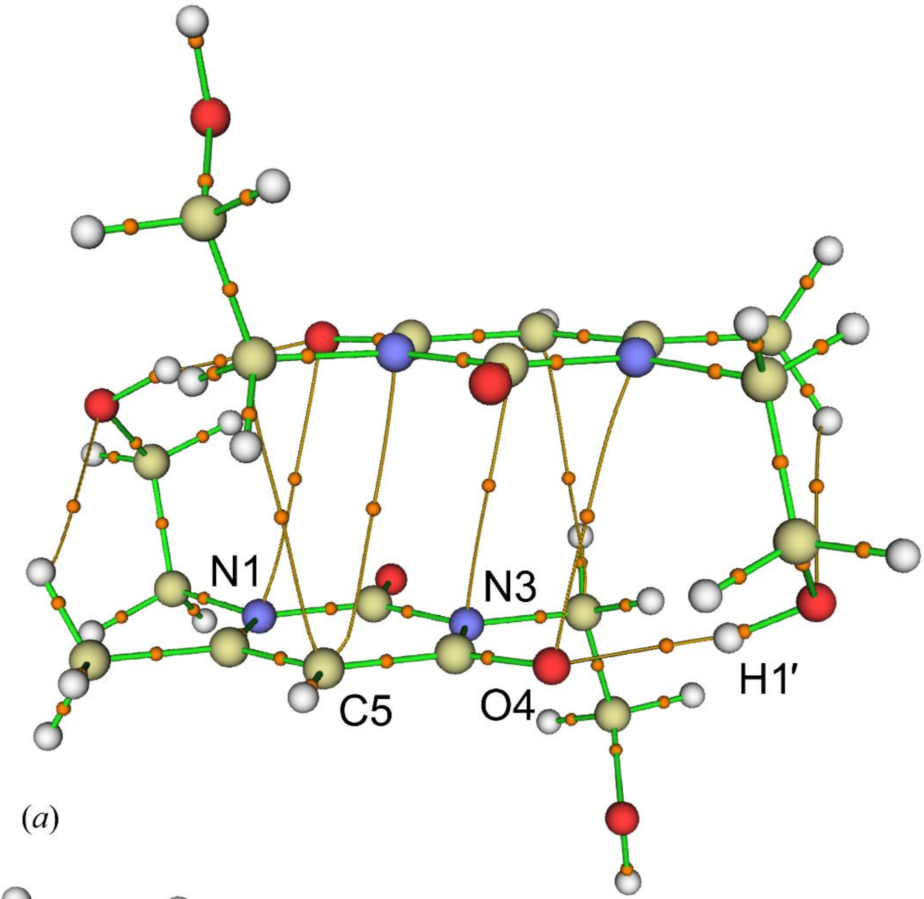

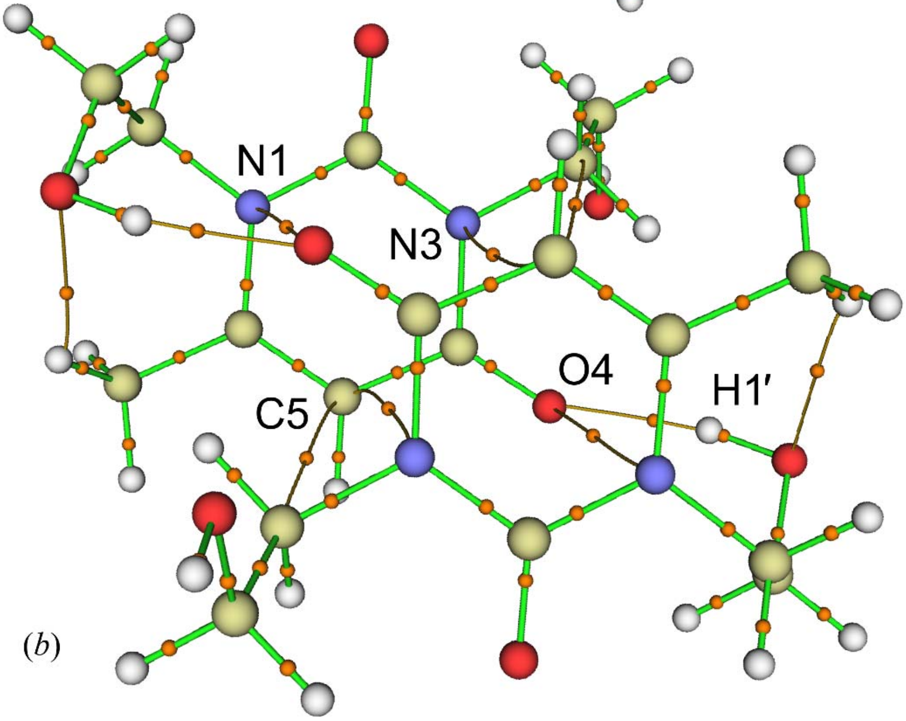

Figure S4 Two different views of the optimized (ωB97X/pob-TZVP-rev2) hydrogen-bonded, π-stacked dimer of the 1,3-bis(2-hydroxyethyl)-6-methyluracil molecules. Bond critical points and bond paths are shown as orange spheres and green or brown lines.

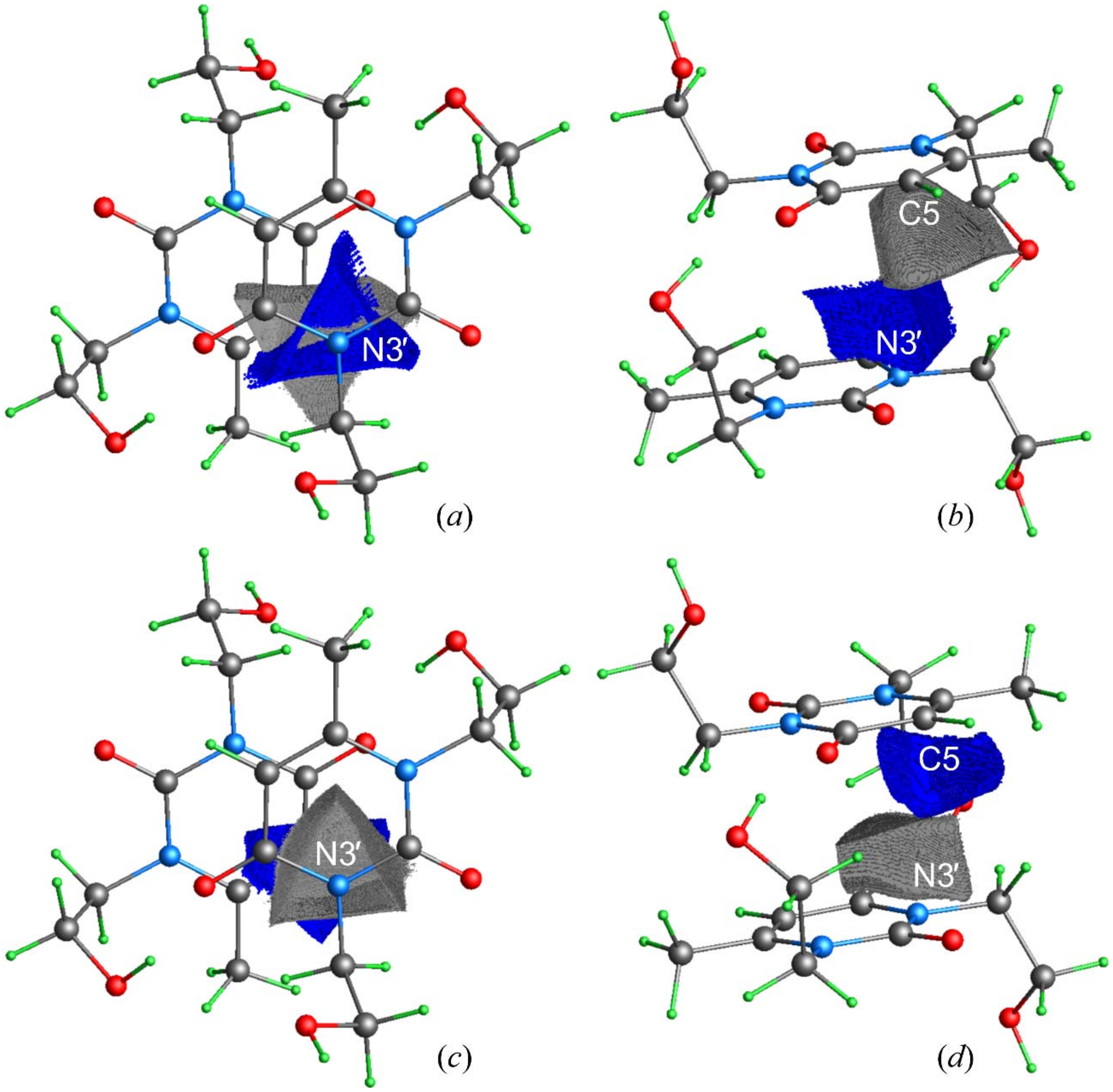

Figure S5 Overlays of the fragments of atomic ρ-basins (gray) and of pseudoatomic φ_{es}-basins (blue) within the intermolecular interaction N3′···C5 of the optimized (ωB97X/pob-TZVP-rev2) hydrogen-bonded, π-stacked dimer of the 1,3-bis(2-hydroxyethyl)-6-methyluracil molecules.

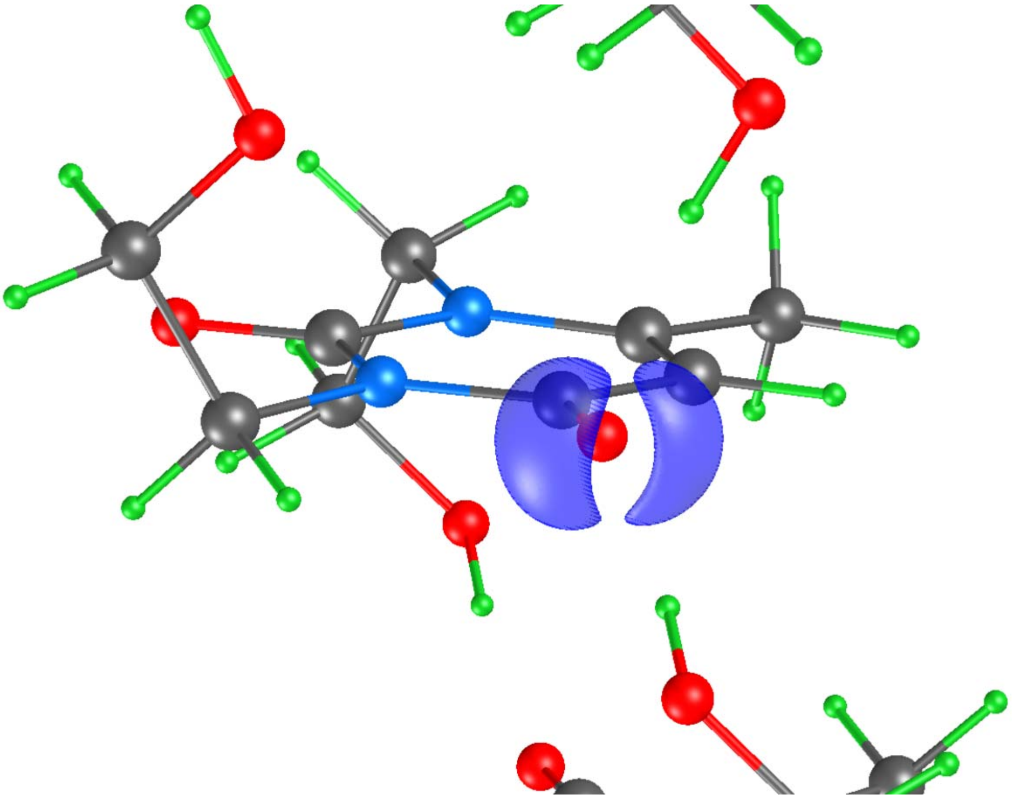

Figure S6 Isosurfaces of the fermionic potential $\varphi_{\mathrm{f}}(\mathbf{r})$ at 0.13 a.u. (blue) in the crystal of 1,3-bis(2-hydroxyethyl)-6-methyluracil around the atom O4, derived from the aspherical pseudo-atom model with parameters fitted to the calculated structure factors (TMM).

Table S1 Optimized geometry of the crystal of 1,3-bis(2-hydroxyethyl)-6-methyluracil at the ωB97X/pob-TZVP-rev2 level.

Atom			*x*/*a*	*y*/*b*	*z*/*c*
1	T	8	–0.2464346257374000	0.3068943878001000	–0.2785094236576000
2	F	8	–0.2535653742626000	–0.1931056121999000	–0.2214905763424000
3	F	8	0.2464346257374000	–0.3068943878001000	0.2785094236576000
4	F	8	0.2535653742626000	0.1931056121999000	0.2214905763424000
5	T	8	–0.2521944939891000	0.0506492200627300	–0.4996845403381000
6	F	8	–0.2478055060109000	–0.4493507799373000	–0.0003154596618528
7	F	8	0.2521944939891000	–0.0506492200627300	0.4996845403381000
8	F	8	0.2478055060109000	0.4493507799373000	0.0003154596618529
9	T	8	–0.2685262565743000	0.2045532818015000	0.2669259111640000
10	F	8	–0.2314737434257000	–0.2954467181985000	0.2330740888360000
11	F	8	0.2685262565743000	–0.2045532818015000	–0.2669259111640000
12	F	8	0.2314737434257000	0.2954467181985000	–0.2330740888360000
13	T	8	–0.4514521498283000	–0.4455879984223000	0.3563317590273000
14	F	8	–0.0485478501716800	0.0544120015776900	0.1436682409727000
15	F	8	0.4514521498283000	0.4455879984223000	–0.3563317590273000
16	F	8	0.0485478501716800	–0.0544120015776900	–0.1436682409727000
17	T	7	–0.1761511219838000	0.3377509595273000	–0.4525677792857000
18	F	7	–0.3238488780162000	–0.1622490404727000	–0.0474322207143100
19	F	7	0.1761511219838000	–0.3377509595273000	0.4525677792857000
20	F	7	0.3238488780162000	0.1622490404727000	0.0474322207143100
21	T	7	–0.3556564710969000	0.3000208262878000	0.4284358651618000
22	F	7	–0.1443435289031000	–0.1999791737122000	0.0715641348381600
23	F	7	0.3556564710969000	–0.3000208262878000	–0.4284358651618000
24	F	7	0.1443435289031000	0.1999791737122000	–0.0715641348381600
25	T	6	–0.2610839268805000	0.2201686971327000	0.4926722913539000
26	F	6	–0.2389160731195000	–0.2798313028673000	0.0073277086460690

27	F	6	0.2610839268805000	–0.2201686971327000	–0.4926722913539000
28	F	6	0.2389160731195000	0.2798313028673000	–0.0073277086460680
29	T	6	–0.3686972569941000	0.4903582396745000	0.4165331129851000
30	F	6	–0.1313027430059000	–0.0096417603254740	0.0834668870149500
31	F	6	0.3686972569941000	–0.4903582396745000	–0.4165331129851000
32	F	6	0.1313027430059000	0.0096417603254740	–0.0834668870149500
33	T	6	–0.2832052228394000	–0.3969218425591000	0.4772252829719000
34	F	6	–0.2167947771606000	0.1030781574409000	0.0227747170281100
35	F	6	0.2832052228394000	0.3969218425591000	–0.4772252829719000
36	F	6	0.2167947771606000	–0.1030781574409000	–0.0227747170281100
37	T	6	–0.1905594703922000	–0.4716542801478000	–0.4593756781677000
38	F	6	–0.3094405296078000	0.0283457198521800	–0.0406243218323200
39	F	6	0.1905594703922000	0.4716542801478000	0.4593756781677000
40	F	6	0.3094405296078000	–0.0283457198521800	0.0406243218323200
41	T	6	–0.0678312652912600	0.2453758370740000	–0.3885021580534000
42	F	6	–0.4321687347087000	–0.2546241629260000	–0.1114978419466000
43	F	6	0.0678312652912600	–0.2453758370740000	0.3885021580534000
44	F	6	0.4321687347087000	0.2546241629260000	0.1114978419466000
45	T	6	–0.1667767031015000	0.1663862217024000	–0.3221505958724000
46	F	6	–0.3332232968985000	–0.3336137782976000	–0.1778494041276000
47	F	6	0.1667767031015000	–0.1663862217024000	0.3221505958724000
48	F	6	0.3332232968985000	0.3336137782976000	0.1778494041276000
49	T	6	–0.4529725358869000	0.1726482406470000	0.3735067052557000
50	F	6	–0.0470274641131200	–0.3273517593530000	0.1264932947443000
51	F	6	0.4529725358869000	–0.1726482406470000	–0.3735067052557000
52	F	6	0.0470274641131200	0.3273517593530000	–0.1264932947443000
53	T	6	–0.3460832972994000	0.0720249925153600	0.3158990940825000
54	F	6	–0.1539167027006000	–0.4279750074846000	0.1841009059175000
55	F	6	0.3460832972994000	–0.0720249925153600	–0.3158990940825000

56	F	6	0.1539167027006000	0.4279750074846000	–0.1841009059175000
57	T	6	–0.1042570561553000	–0.3514634931825000	–0.3958294718655000
58	F	6	–0.3957429438447000	0.1485365068175000	–0.1041705281345000
59	F	6	0.1042570561553000	0.3514634931825000	0.3958294718655000
60	F	6	0.3957429438447000	–0.1485365068175000	0.1041705281345000
61	T	1	–0.3524601893091000	0.3455454131960000	–0.3078322385798000
62	F	1	–0.1475398106909000	–0.1544545868040000	–0.1921677614202000
63	F	1	0.3524601893091000	–0.3455454131960000	0.3078322385798000
64	F	1	0.1475398106909000	0.1544545868040000	0.1921677614202000
65	T	1	–0.1924975332665000	0.1411546534124000	0.2326341991518000
66	F	1	–0.3075024667335000	–0.3588453465876000	0.2673658008482000
67	F	1	0.1924975332665000	–0.1411546534124000	–0.2326341991518000
68	F	1	0.3075024667335000	0.3588453465876000	–0.2673658008482000
69	T	1	–0.2945640001718000	–0.2479846516261000	0.4710085715715000
70	F	1	–0.2054359998282000	0.2520153483739000	0.0289914284285200
71	F	1	0.2945640001718000	0.2479846516261000	–0.4710085715715000
72	F	1	0.2054359998282000	–0.2520153483739000	–0.0289914284285200
73	T	1	0.0251416988517900	0.3445283608628000	–0.3630641728482000
74	F	1	0.4748583011482000	–0.1554716391372000	–0.1369358271518000
75	F	1	–0.0251416988517900	–0.3445283608628000	0.3630641728482000
76	F	1	–0.4748583011482000	0.1554716391372000	0.1369358271518000
77	T	1	0.0005099255160784	0.1342862058390000	–0.4167448212116000
78	F	1	0.4994900744839000	–0.3657137941610000	–0.0832551787884200
79	F	1	–0.0005099255160784	–0.1342862058390000	0.4167448212116000
80	F	1	–0.4994900744839000	0.3657137941610000	0.0832551787884200
81	T	1	–0.0752453512793900	0.0958859023450600	–0.2800617450679000
82	F	1	–0.4247546487206000	–0.4041140976549000	–0.2199382549321000
83	F	1	0.0752453512793900	–0.0958859023450600	0.2800617450679000
84	F	1	0.4247546487206000	0.4041140976549000	0.2199382549321000

85	T	1	–0.2595186396917000	0.0634940389340000	–0.3456006982393000
86	F	1	–0.2404813603083000	–0.4365059610660000	–0.1543993017607000
87	F	1	0.2595186396917000	–0.0634940389340000	0.3456006982393000
88	F	1	0.2404813603083000	0.4365059610660000	0.1543993017607000
89	T	1	0.4860161680240000	0.0712441615691400	0.4104110241849000
90	F	1	0.0139838319759900	–0.4287558384309000	0.0895889758150700
91	F	1	–0.4860161680240000	–0.0712441615691400	–0.4104110241849000
92	F	1	–0.0139838319759900	0.4287558384309000	–0.0895889758150700
93	T	1	0.4495145882111000	0.2547171150497000	0.3404052663738000
94	F	1	0.0504854117888900	–0.2452828849503000	0.1595947336262000
95	F	1	–0.4495145882111000	–0.2547171150497000	–0.3404052663738000
96	F	1	–0.0504854117888900	0.2452828849503000	–0.1595947336262000
97	T	1	–0.2494313176063000	–0.0139310201449100	0.3485522651015000
98	F	1	–0.2505686823937000	0.4860689798551000	0.1514477348985000
99	F	1	0.2494313176063000	0.0139310201449100	–0.3485522651015000
100	F	1	0.2505686823937000	–0.4860689798551000	–0.1514477348985000
101	T	1	–0.4314284442535000	–0.0205693539503100	0.2795269727249000
102	F	1	–0.0685715557464600	0.4794306460497000	0.2204730272751000
103	F	1	0.4314284442535000	0.0205693539503100	–0.2795269727249000
104	F	1	0.0685715557464600	–0.4794306460497000	–0.2204730272751000
105	T	1	–0.1529635042120000	–0.3792100103386000	–0.3375418600741000
106	F	1	–0.3470364957880000	0.1207899896614000	–0.1624581399259000
107	F	1	0.1529635042120000	0.3792100103386000	0.3375418600741000
108	F	1	0.3470364957880000	–0.1207899896614000	0.1624581399259000
109	T	1	0.0331076177189100	–0.3747202501597000	–0.3890947915685000
110	F	1	0.4668923822811000	0.1252797498403000	–0.1109052084315000
111	F	1	–0.0331076177189100	0.3747202501597000	0.3890947915685000
112	F	1	–0.4668923822811000	–0.1252797498403000	0.1109052084315000
113	T	1	–0.1280084928719000	–0.2064408356193000	–0.4115295654610000

114	F	1	–0.3719915071281000	0.2935591643807000	–0.0884704345390300
115	F	1	0.1280084928719000	0.2064408356193000	0.4115295654610000
116	F	1	0.3719915071281000	–0.2935591643807000	0.0884704345390300

"T" marks the atoms belonging to the asymmetric cell. The fractional coordinates *x*/*a*, *y*/*b*, and *z*/*c* are given in angstroms.

Table S2 Optimized geometry of the hydrogen-bonded dimer of 1,3-bis(2-hydroxyethyl)-6-methyluracil at the *ω*B97X-D/aug-cc-pVTZ level.

Center number	Atomic number	*x*	*y*	*z*
1	1	1.565820	–4.332110	0.688453
2	1	–0.057651	–4.859086	1.170879
3	8	3.604658	1.996683	0.713193
4	8	2.607311	–2.047961	1.322048
5	8	0.192773	–4.508430	–0.861991
6	8	–1.000504	–1.436019	–1.379358
7	7	2.816507	–0.618376	–0.431706
8	7	0.814847	–1.812158	–0.047102
9	6	2.110329	–1.524707	0.346373
10	6	0.174062	–1.208388	–1.125865
11	6	0.983137	–0.299467	–1.880978
12	6	2.251627	–0.012961	–1.527715
13	6	4.157843	–0.283253	0.054676
14	6	4.134938	0.772091	1.155035
15	6	0.087645	–2.780417	0.767995
16	6	0.499340	–4.210739	0.485259
17	6	3.068914	0.959361	–2.315175
18	1	2.662783	2.000249	0.936685
19	1	0.576023	–5.353802	–1.090809
20	1	0.524084	0.168285	–2.735559

21	1	5.166190	0.939897	1.474111
22	1	3.575861	0.387122	2.009862
23	1	0.273747	–2.554286	1.815204
24	1	–0.967074	–2.646709	0.550232
25	1	3.433012	1.759314	–1.671505
26	1	2.459180	1.380367	–3.109445
27	1	4.749268	0.065834	–0.785609
28	1	4.607584	–1.196325	0.436652
29	1	3.929449	0.467415	–2.770713
30	1	–4.748459	–0.067578	0.786976
31	1	–4.608098	1.195112	–0.434892
32	1	–3.927433	–0.469031	2.771660
33	8	–3.603283	–1.997187	–0.712901
34	8	–2.608744	2.047897	–1.321284
35	8	–0.194082	4.509101	0.861418
36	8	1.000845	1.437169	1.377996
37	7	–2.81625	0.617825	0.432272
38	7	–0.8154	1.812632	0.046776
39	6	–2.11095	1.52466	–0.34602
40	6	–0.17375	1.209021	1.125134
41	6	–0.98198	0.299617	1.880553
42	6	–2.25051	0.012516	1.527873
43	6	–4.15766	0.282144	–0.05349
44	6	–4.13467	–0.77284	–1.15418
45	6	–0.08905	2.781253	–0.76867
46	6	–0.50124	4.211381	–0.48569
47	6	–3.06686	–0.9604	2.315568
48	1	–2.66168	–2.00031	–0.93764
49	1	–0.57483	5.355798	1.089532

50	1	–0.52226	–0.16803	2.734832
51	1	–5.16597	–0.9412	–1.47282
52	1	–3.57626	–0.38728	–2.00918
53	1	–0.27561	2.555053	–1.81578
54	1	0.965829	2.648017	–0.55138
55	1	–3.43086	–1.76041	1.671907
56	1	–2.45649	–1.38125	3.109431
57	1	–1.56791	4.332265	–0.68826
58	1	0.055055	4.859997	–1.17161

The *x*, *y*, and *z* coordinates are given in angstroms.
